# Long non-coding RNA Myd88 promotes growth and metastasis in hepatocellular carcinoma via regulating Myd88 expression through H3K27 modification

**DOI:** 10.1038/cddis.2017.519

**Published:** 2017-10-12

**Authors:** Xiaoliang Xu, Yin Yin, Junwei Tang, Yu Xie, Zhuo Han, Xudong Zhang, Qiaoyu Liu, Xihu Qin, Xinli Huang, Beicheng Sun

**Affiliations:** 1 Liver Transplantation Center of the First Affiliated Hospital and State Key Laboratory of Reproductive Medicine, Nanjing Medical University, Nanjing, Jiangsu Province, PR China; 2 Department of General Surgery, Huai'an First People's Hospital, Nanjing Medical University, Huai'an, Jiangsu Province, PR China; 3 The Affiliated Changzhou NO.2 People’s Hospital of Nanjing Medical University, Changzhou, Jiangsu Province, PR China

## Abstract

Enhanced Myd88 expression has been found in various parenchymal tumors especially in hepatocellular carcinoma with little mechanism of its upregulation known. A lot of long non-coding RNAs are reported to regulate the protein-coding genes which have location association through various mechanisms. In our study we confirmed a new long non-coding RNA Myd88 aberrant upregulated in HCC located upstream of Myd88 and verified a positive regulation relationship between them indicating that Lnc-Myd88 might participate in the enhanced expression of Myd88 in HCC. The gain- and loss-of-function analysis revealed that Lnc-Myd88 could promote the proliferation and metastasis of HCC both *in vitro* and *in vivo*. In addition, ChIP assays demonstrated that Lnc-Myd88 might increase Myd88 expression through enhancing H3K27Ac in the promoter of Myd88 gene, thus resulting in the activation of both NF-*κ*B and PI3K/AKT signal pathways. In conclusion, we proposed that Lnc-Myd88 might serve as a novel diagnosis and therapeutic target for HCC.

Hepatocellular carcinoma (HCC) is one of the most common cancers and the third leading cause of cancer-related death worldwide,^1, 2^ with half of these occurring in china.^3, 4^ Although the great advancements in therapeutic methods, such as liver resection, liver transplantation, chemotherapy and radiotherapy have been acquired, the prognosis of patients with HCC still remains poor.^5^ HCC has been identified with a preference for intrahepatic or extrahepatic metastasis leading to a high recurrence rate after surgery.^6, 7, 8^ It is well known that the molecular mechanisms underlying the oncogenesis and metastasis of HCC are not well explored, and there still lays a great barrier before us in exploring new biomarkers which may contribute to improve the diagnosis and prognosis of HCC.^9^

Long non-coding RNAs (lncRNAs) are defined as a subgroup of non-coding RNAs with no or limited protein-coding capability which are composed of greater than 200 nucleotides.^10, 11^ Accumulating researchers prove that lncRNAs display multiple critical functions in the biological developments of various tumors through complicated mechanisms, including oncogenesis and tumor progression.^10, 11, 12, 13^ Notably, lots of lncRNAs have been found to play a part in HCC, including HULC, HEIH, MVIH, HOTAIR and so on.^14, 15, 16, 17^ The mechanisms of lncRNAs have been widely investigated, including modulating the transcription and post-transcription process of genes, controlling gene expression through chromatin remodeling, adjusting protein function or localization.^18^ In addition, researchers have discovered that lncRNAs could participate in gene expression through epigenetic regulation including methylation, acetylation and ubiquitination.^19^

With the advancement of sequencing and annotation of whole genomes, a novel study reveals that almost >65% lncRNAs located within 10 kb of known protein-coding genes which is known as the 'Flank10kb' theory, suggesting that there may exist *cis*-acting or *trans*-regulatory relationships between lncRNAs and known genes.^20, 21^ Additionally, a larger part of lncRNAs shared the bidirectional promoter with protein-coding genes, which indicates that lncRNAs may modulate the expression of neighbor protein-coding genes in a bidirectional transcription fashion.^22, 23^ Thereby, to explore the interaction of location-associated lncRNAs and protein-coding genes, we conducted a systematic search using a lot of databases including PubMed, RNAdb, LNCipedia, UCSC Genome Browser and lncrnadb. Finally, a recognized lncRNA named LOC105377033 was chosen as the candidate lncRNA located upstream of Myd88 with a distance of 20kp, and presented a reverse transcription direction with Myd88. We further named it as Lnc-Myd88. Myd88 is defined as a carcinogenic gene in HCC, promoting growth and metastasis of human HCC,^12, 24, 25^ which can serve as a prognostic and therapeutic target factor for HCC,^26^ while the function of Lnc-Myd88 in HCC still remains unclear.

In this study, we tried to figure out the role of Lnc-Myd88 in HCC, including hepatocarcinogenesis and progression. In addition, we attempted to identify the mechanism by which Lnc-Myd88 regulate the expression of Myd88.

## Results

### Lnc-Myd88 is upregulated with a high correlation with Myd88 in HCC tissues and correlated with poor prognosis

**Figure 1:**
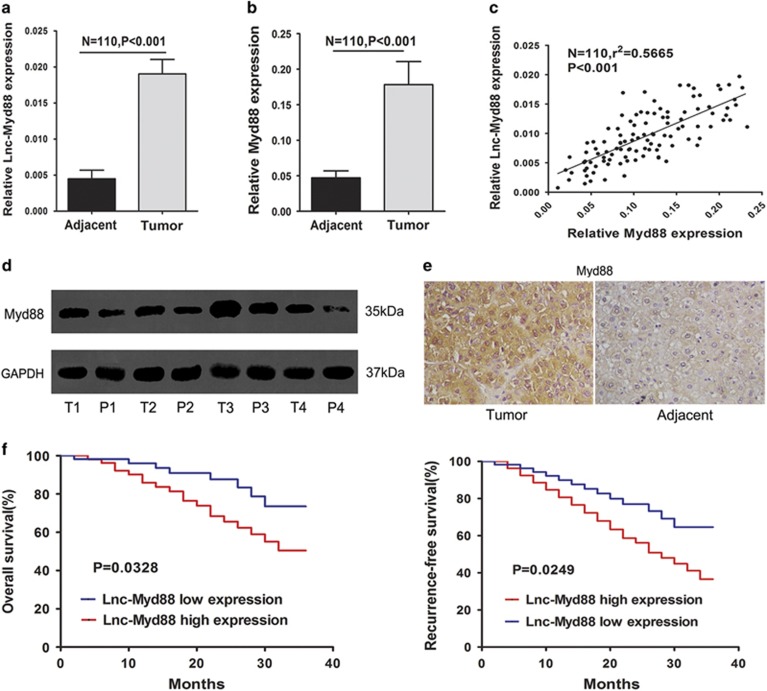
Lnc-Myd88 is upregulated with a high correlation with Myd88 in hepatocellular carcinoma tissues and correlated with poor prognosis. (**a**) Ectopic expression of Lnc-Myd88 in HCC tumor tissues and corresponding adjacent normal liver tissues were detected by quantitative real-time PCR normalized to GAPDH (*N*=110, *P*<0.001). (**b**) Enhanced expression of Myd88 in HCC tissues compared with the adjacent normal liver tissues were detected by quantitative real-time PCR normalized to GAPDH (*N*=110, *P*<0.001). (**c**) A positive correlation between expression levels of Myd88 and Lnc-Myd88 determined by Pearson analysis (*N*=110, *r*^2^=0.5665,  *P*<0.001). (**d** and **e**) Relative expression level of Myd88 protein in HCC tissues and adjacent tissues were tested by western blotting and immunohistochemical assays (original magnification × 200). (**f**) According to the median value, patients were divided into two groups according to Lnc-Myd88 expression in HCC tissues. The log-rank test was used to calculated the overall survival and recurrence-free survival of patients

On the basis of the bioinformatics analyses, we found that Lnc-Myd88 was located upstream of Myd88, with a spacing of about 20 kb. Firstly, to explore whether Lnc-Myd88 interrelated with HCC, we detected its expression in tumor tissues and corresponding adjacent normal liver tissues. Lnc-Myd88 was significantly upregulated in HCC tissues compared with adjacent normal liver tissues (Figure 1a), indicating that Lnc-Myd88 might be involved in the nosogenesis of HCC. Since Lnc-Myd88 was a *de-novo* lncRNA, we performed experiments to figure out its protein-coding ability and subcellular localization in liver tumor tissues and cell lines. To determine whether the transcript of Lnc-Myd88 might encode proteins, we consulted the Coding Protein Calculator (http://cpc.cbi.pku.edu.cn/) (Supplementary Figure S1A). Comparing with ANRIL, a well-studied non-coding RNA, Lnc-Myd88 was more inclined to be a non-coding RNA. According to the results of RT-PCR amplified with separated cytoplasm RNA and nuclear RNA, we discovered that Lnc-Myd88 was mainly located in the nucleus of Huh7 and SMMC-7721 cell lines (Supplementary Figure S1B).

Myd88 was known as a tumor promoter, which has been found in various cancers and was related to tumor development, especially in HCC, Myd88 was shown to promote tumor cell proliferation, invasion, metastasis and correlated with prognosis of HCC patients. The ectopic expression of Myd88 was confirmed by qRT-PCR, western blotting and IHC in our study (Figures 1b). To detect whether there was a correlation between the expression level of Lnc-Myd88 and Myd88 in HCC tumors, we conducted qRT-PCR in 110 pairs of tissues samples. According to the results of qRT-PCR, we conducted the Pearson correlation analysis to detect the correlation between the expression level of Myd88 and Lnc-Myd88. The results indicated a positive correlation with a *P*-value of <0.001 and *r*^2^=0.5665, suggesting there was a positive regulation relationship between Lnc-Myd88 and Myd88 (Figure 1c). To understand the significance of the aberrant expression of Lnc-Myd88 in HCC, we investigated the potential associations between the expression of Lnc-Myd88 and patients’ clinicopathological features. As presented in Table 1, the median value was set as the cutoff to classify the expression level of Lnc-Myd88, patients were then divided into high-Lnc-Myd88 and low-Lnc-Myd88 groups. Statistical analysis showed that a higher expression level of Lnc-Myd88 in HCC was associated with increased tumor size, worse tumor differentiation grade and metastasis. However, other clinical parameters including age, gender, HBsAg, serum AFP level, cirrhosis and tumor number were not found correlations with Lnc-Myd88 expression. Furthermore, survival analysis showed that patients with high Lnc-Myd88 expression in HCC had significantly worse prognosis than those with low Lnc-Myd88 expression, with a lower overall survival rate and shorter non-recurrence period (Figure 1f).

### Lnc-Myd88 promotes cell proliferation and metastasis *in vitro*

**Figure 2:**
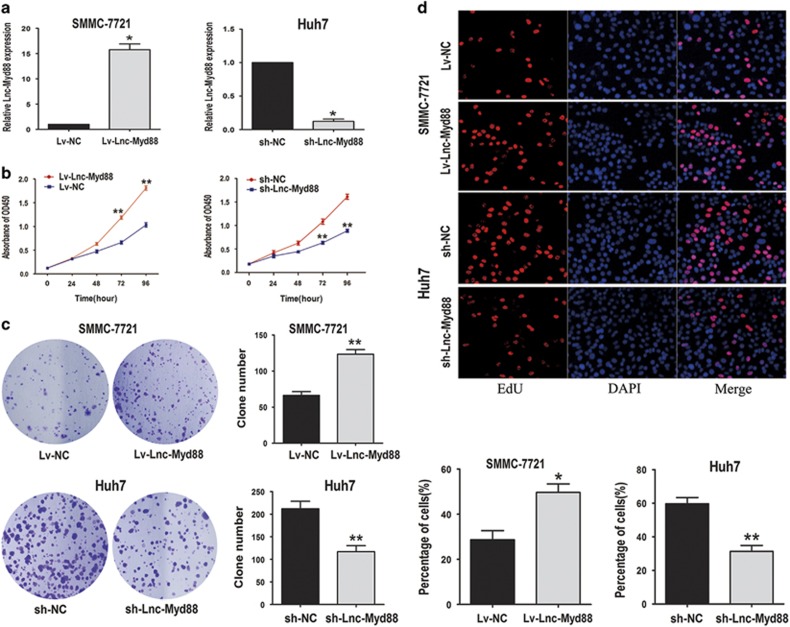
Lnc-Myd88 promotes HCC cells proliferation *in vitro*. (**a**) The transfection efficiency of ectopic expression and gene silencing of Lnc-Myd88 in SMMC-7721 and Huh7 cells was determined by qRT-PCR. (**b**) Proliferation ability was detected by CCK8 assay, overexpression of Lnc-Myd88 promoted SMMC-7721 cells proliferation, whereas knockdown of Lnc-Myd88 inhibited Huh7 cells proliferation. (**c**) Colony formation assays were performed on differently treated HCC cells for 2 weeks, representative graphs are shown. (**d**) EdU immunofluorescence staining confirmed the function of Lnc-Myd88 on HCC cells proliferation. Original magnification × 200. Stable overexpression of Lnc-Myd88 increased the proliferation of SMMC-7721 cells while knockdown of Lnc-Myd88 decreased the proliferation of Huh7 cells. All experiments were performed in triplicate and presented as the mean±S.E.M. (*P<0.05, **P<0.01)

**Figure 3:**
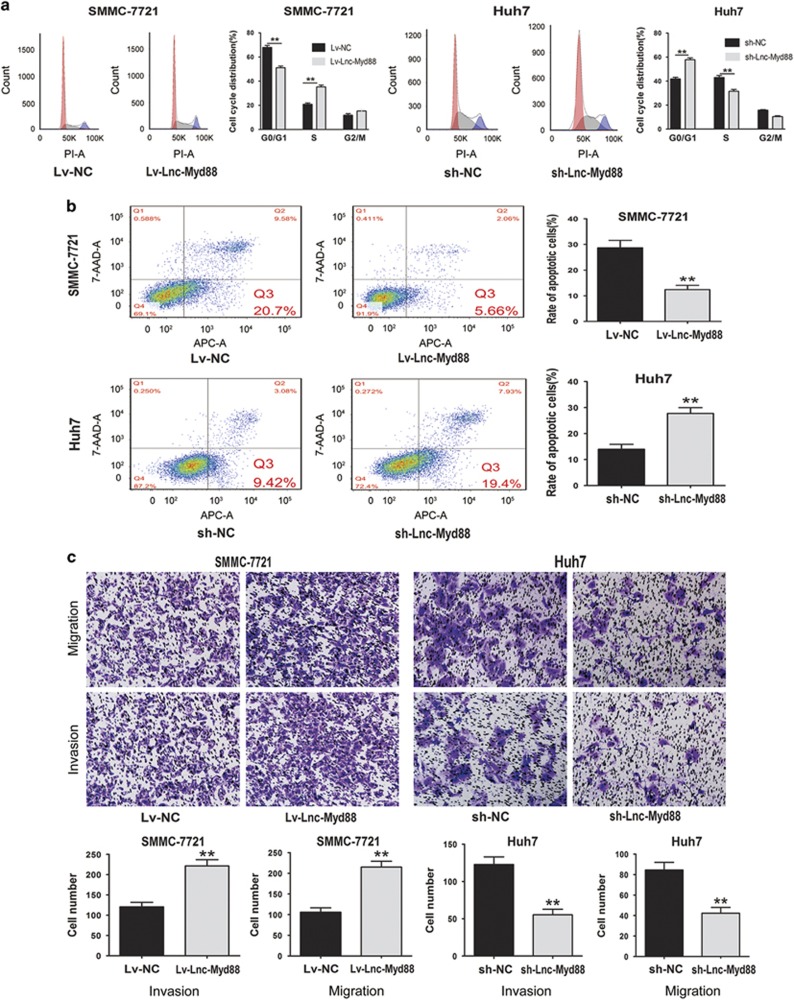
Lnc-Myd88 regulates hepatoma cells cell cycle, cell apoptosis and cell migration *in vitro*. (**a**) Cell-cycle analysis of SMMC-7721 cells stable overexpressing Lnc-Myd88 and Huh7 cells stable silenced Lnc-Myd88 expression was conducted by flow cytometry. The distribution of the cell cycle was shown in the graphs. (**b**) Cells were cultured with complete medium with 0.5 mM peroxide overnight, and cell apoptosis rate was detected by flow cytometry using the Annexin V-APC/7-AAD staining kit. The bar graph shows the percentage of apoptotic cells. (**c**) Invasion and migration assay of Lnc-Myd88 overexpressed and silenced cells. The bar graph shows the number of cells migrated or invaded through the membrane. Original magnification × 200. All experiments were performed in triplicate and presented as the mean±S.E.M. (*P<0.05, **P<0.01)

To investigate the biological functions of Lnc-Myd88 *in vitro*, firstly, we detected the endogenous level of Lnc-Myd88 in HCC cell lines, as presented in Supplementary Figure S1C, there was a different distribution for the expression of Lnc-Myd88 in HCC cell lines, among which SMMC-7721 showed the lowest level while Huh7 indicating a higher endogenous expression. According to the loss-and-gain function assay reported by various researchers, we selected SMMC-7721 cell line to construct Lnc-Myd88 overexpression model and Huh7 cell line to construct Lnc-Myd88 knockdown model (Figure 2a). According to the results of clone formation assays, compared with the control groups, we found there were more clones formed in the Lnc-Myd88 ectopically overexpressed groups, whereas knockdown of Lnc-Myd88 obviously inhibited the clone formation ability in Huh7 cells (Figure 2c). Furthermore, in the CCK8 assays, we found that Lv-Lnc-Myd88-SMMC-7721 cells presented an increased proliferation than Lv-NC-SMMC-7721 cells, whereas the proliferation of sh-Lnc-Myd88-Huh7 cells was suppressed, indicating that Lnc-Myd88 might promote the HCC cells growth (Figure 2b). Besides, to confirm the function of Lnc-Myd88 in cell growth, we conducted the EdU staining assay (Figure 2d). Stable ectopically overexpression of Lnc-Myd88 increased the numbers of EDU-positive nuclei of SMMC-7721 cells than the controls, In addition, comparing with the controls, stable knockdown of Lnc-Myd88 reduced the numbers of EdU-positive nuclei of Huh7 cells. We assumed whether Lnc-Myd88 accelerated HCC cells proliferation by promoting cell-cycle progression and inhibiting cell apoptosis. Next, we utilized the FACS technology to explore whether Lnc-Myd88 could make a difference to cell cycle and cell apoptosis. The cell-cycle experiments showed a reduction in the G0/G1 population and an elevation in the S-phase population in Lnc-Myd88-overexpressed SMMC-7721 cells, whereas Lnc-Myd88 knockdown had opposite effects in Huh7 cells (Figure 3a). Furthermore, the results of cell apoptosis assays indicated that Lnc-Myd88 overexpression could reduce cell apoptosis in SMMC-7721 cells, whereas sh-Lnc-Myd88-Huh7 cells got an apoptosis enhancement compared with the controls (Figure 3b). All of these results proved that Lnc-Myd88 promoted HCC cells growth and proliferation.

The correlation analysis between Lnc-Myd88 and clinicopathological characteristics indicated that Lnc-Myd88 might promote HCC metastasis. To investigate whether Lnc-Myd88 had a direct functional role in cell invasion and migration, we conducted transwell assays. The results showed that the migration and invasion abilities of SMMC-7721 cells were significantly increased when the Lnc-Myd88 was overexpressed, while Lnc-Myd88 knockdown significantly reduced cell migration and invasion in Huh7 cells (Figure 3c). On the basis of results above, we proposed that Lnc-Myd88 promoted the migration and invasion of HCC cells.

### Lnc-Myd88 enhances tumor growth and metastasis *in vivo*

**Figure 4:**
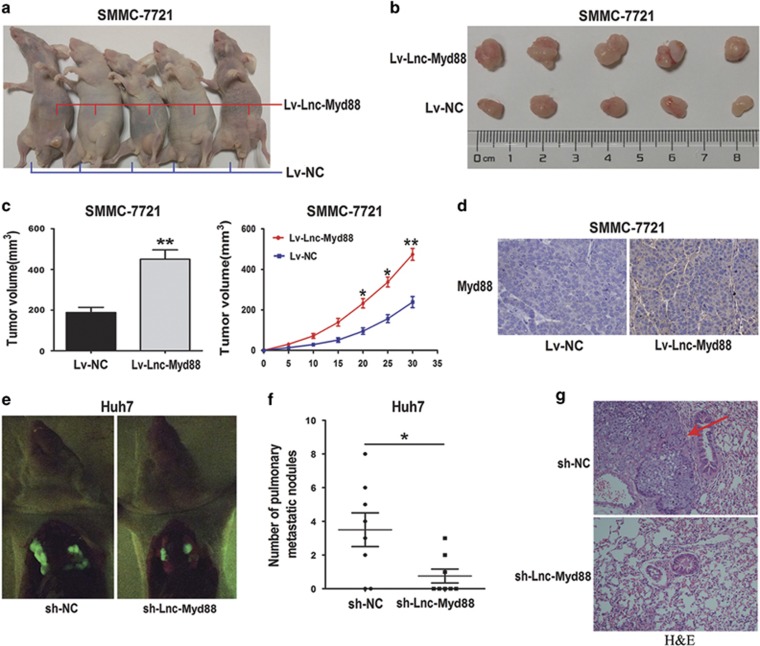
Lnc-Myd88 enhances tumor growth and metastasis *in vivo*. (**a–c**) BAB/c nude mice (6 weeks of age) were subcutaneous transplantated with SMMC-7721 cells, Lv-Lnc-Myd88-SMMC-7721 cells (1 × 10^7^) in the right groin and Lv-NC SMMC-7721 (1 × 10^7^) cells in the left groin (5 mice in each group). The volume of tumors was calculated every 5 days after transplantation and mice were killed 30 days after implantation. Lnc-Myd88 strengthen the tumor growth of SMMC-7721 cells in nude mice. The volume of each tumor was calculated as the length × width^2^ × 0.5. (**d**) Myd88 expression level in the tumor samples determined by IHC. Original magnification × 200. (**e**) In the tail vein xenograft model, mice (8 in each group) were injected with Huh7 cells (1 × 10^7^ suspended in 200 *μ*l PBS) through the tail vein and killed 5 weeks later, lung metastasis were investigated in each group respectively by an *in vivo* fluorescence imaging system. (**f**) Compared with the Lnc-Myd88 knockdown group (three mice presented lung colonization), six mice presented lung colonization with more and larger tumors in the control group. (**g**) All the results of lung colonization were validated by the histological examination (H&E). Original magnification × 200 (*P<0.05, **P<0.01)

To further investigate the functional role of Lnc-Myd88 *in vivo*, we established a xenotransplantation model in which nude mouse were subcutaneously injected with Lnc-Myd88 ectopically overexpressed and controlled SMMC-7721 cells (Figures 4a and b). We found that the xenografts produced from Lnc-Myd88-overexpressed cells grown faster than the controls, and the final tumor volume and weight were larger than the controls (Figure 4c). We also detected the Myd88 expression level in the tumor samples above by IHC, and the Myd88 expression was increased in Lv-Lnc-Myd88-SMMC-7721 cells compared with the controls (Figure 4d). Next, to investigate the lung metastasis changes of HCC cells, we built a tail vein xenograft model. We set up two groups with eight mice in each group, one group was injected with Lnc-Myd88 knockdown Huh7 cells and the other was the controls which were transfected with GFP (Figure 4e). The results revealed that some lung metastasis nodules were formed in the control group with six mice presented lung colonization at the end of the experiment, while only three mice established the lung colonization in the knockdown group with fewer and smaller tumors than the control group (Figure 4f). All the results of lung colonization were validated by the histological examination (Figure 4g). Taken together, these results proved that Lnc-Myd88 promoted both growth and metastasis of HCC tumors *in vivo*. Next, we explored the mechanism of Lnc-Myd88 in HCC.

### Lnc-Myd88 induces an upregulation of Myd88 by enhanced acetylation of the promoter of Myd88

**Figure 5:**
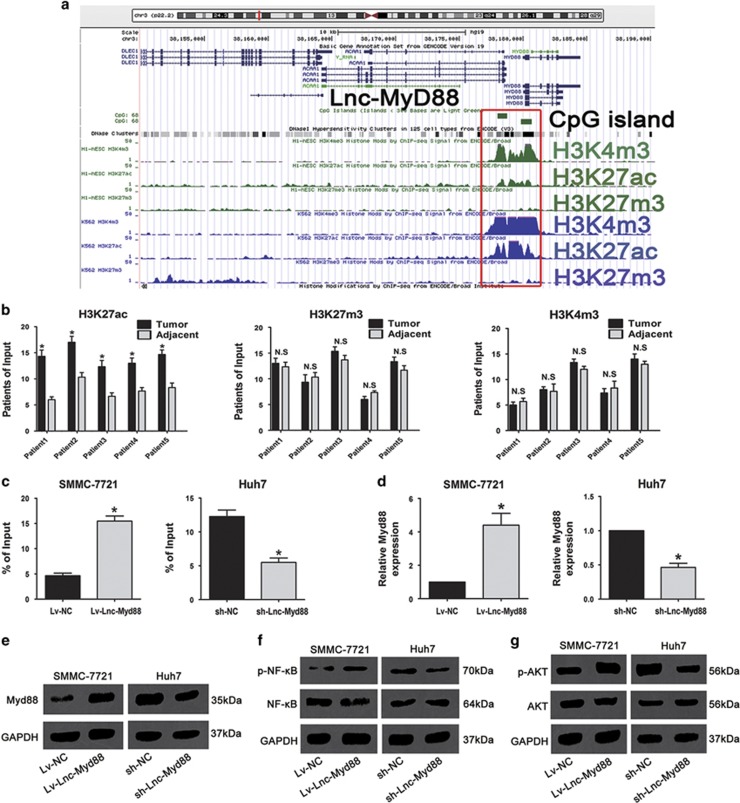
Lnc-Myd88 induces an upregulation of Myd88 by enhanced acetylation of the promoter of Myd88 and then activates NF-*κ*B and PI3K/AKT signal pathways. (**a**) Enrichment prediction of H3K4m3, H3K27Ac and H3K27m3 in the promoter region of Myd88 through ENCODE database. (**b**) The enrichment of H3K4m3, H3K27Ac and H3K4m3 in the promoter region of Myd88 in HCC patients. (**c**) The alteration of the enrichment of H3K27Ac in the promoter region of Myd88 in HCC cells treated with Lnc-Myd88. (**d** and **e**) The alteration of mRNA and protein level of Myd88 in HCC cells induced by Lnc-Myd88. GAPDH was used as a loading control. (**f** and **g**) The levels of NF-*κ*B, p-NF-*κ*B, AKT, p-AKT and GAPDH were examined by western blotting in SMMC-7721 and Huh7 cells treated with Lnc-Myd88. The experiments were performed in triplicate; the data are expressed as the mean±S.E.M. (*P<0.05, **P<0.01)

In consideration of the significant tumor-promoting function of Lnc-Myd88 *in vitro* and *in vivo*, we wondered whether the effect of Lnc-Myd88 was related with the neighbor gene Myd88. According to the analysis of ENCODE database, we found that compared with the promoter region of Lnc-Myd88, upstream of the transcriptional start site of Myd88, there were many CpG islands in the promoter region of Myd88. And there were enrichment of H3K27Ac and H3K4m3 but low concentration of H3K27m3 in the promoter region of Myd88 in H1-hESC and K562 cells, indicating that histone modification might participate in the expression of Myd88 in transcriptional regulation (Figure 5a). Hence, we conducted chromatin immunoprecipitation (ChIP) assays to detect the enrichment of H3K27Ac, H3K27m3 and H3K4m3 of the promoter in five paired tissues which were verified with aberrant upregulated Myd88 and Lnc-Myd88 expression in tumors compared with adjacent normal tissues. As presented in the (Figure 5b), we found that the acetylation of H3K27 in the promoter of Myd88 was enriched, while there was no difference of H3K27m3 and H3K4m3 in the promoter of Myd88. Thus, we could draw a hypothesis that Lnc-Myd88 might participate in the acetylation of H3K27 in the promoter of Myd88. To confirm this hypothesis, we tested the enrichment of H3K27Ac in the promoter of Myd88 by ChIP assay, as shown in Figure 5c, upregulation of Lnc-Myd88 resulted in enhanced H3K27Ac of Myd88 whereas downregulation of Lnc-Myd88 decreased the enrichment of H3K27Ac of Myd88. As documented in the previous reports, the enhanced acetylation of H3K27 was an important agonist for gene expression. Next, we used qRT-PCR to detect the mRNA expression level of Myd88 in HCC cells treated with Lnc-Myd88, and found that compared with the controls, there were increased expression of Myd88 in Lv-Lnc-Myd88-SMMC-7721 cells, whereas the expression level of Myd88 was decreased in the sh-Lnc-Myd88-Huh7 cells (Figure 5d). Next, we tested the alternation on the protein level using western blotting and got the same results (Figure 5e). On account of the results of IHC mentioned above, we draw a conclusion that Lnc-Myd88 may regulate Myd88 expression on mRNA and protein level in HCC cells by regulating the enrichment of H3K27Ac in the promoter region of Myd88.

### NF-*κ*B and PI3K/AKT signal pathways are promoted by the upregulation of Myd88 induced by Lnc-Myd88

According to the previous literatures, Myd88 was documented as a tumor promoter in various cancers, especially in HCC, it was shown to promote tumor cell proliferation, invasion, metastasis through activation of NF-*κ*B and PI3K/AKT signal pathways. Since we found that Lnc-Myd88 could promote HCC cells growth and metastasis *in vitro* and *in vivo* and regulated the expression of Myd88 in transcription level through adjusting the enrichment of the H3K27ac of the promoter of Myd88, we wondered whether NF-*κ*B and PI3K/AKT signal pathways were involved in the mechanism of carcinogenic function of Lnc-Myd88. To verify this hypothesis, we tested the expression of these two pathways by western blotting. As shown in Figure 5f, fortunately, overexpression of Lnc-Myd88 greatly strengthen NF-*κ*B activation in SMMC-7721 cells, and knockdown of Lnc-Myd88 was able to significantly inhibit intrinsic NF-*κ*B activity in MyD88 high expression Huh7 cells. Also, we found that upregulation of Lnc-Myd88 greatly enhanced the intrinsic activation of AKT in SMMC-7721 cells, downregulation of Lnc-Myd88 attenuated the activation of AKT in Huh7 cells (Figure 5g). All in all we came a conclusion that Lnc-Myd88 might promote tumor progression through upregulating Myd88 and then activating the NF-*κ*B and PI3K/AKT signal pathways.

## Discussion

Recent years, long non-coding RNAs have been reported as significant regulatory molecules involved in multiple biological processes and various diseases, including HCC.^23^ Of the various and complicated mechanisms of lncRNAs, histone modifications including acetylation, methylation and ubiquitination are emerging as a crucial regulatory mechanism of the interaction between lncRNAs and their neighbor protein-coding genes.^27, 28^ For instance, DNMTs are documented to be recruited by lncRNAs to promote the methylation of CpG islands or modify the trimethylation of H3K27 in the promoter region of a gene. Furthermore, a part of lncRNAs have been demonstrated to perform their function in regulating the acetylation of H3K27ac in the promoter region of genes.^29^ In this study, on account of the complicated molecular mechanisms mentioned above, we attempted to investigate the potential regulation pattern between lncRNAs and neighbor protein-coding genes.^20^

Myd88 has been found overexpressed in various types of parenchymal cancers with little mechanism of its upregulation known. As reported in previous studies, enhanced Myd88 could not only evade apoptotic stimulation and promote cell-cycle progress but also confer tumor cells with strengthened abilities of proliferation, migration and invasion especially in HCC. And the tumor-promoting function of Myd88 was ascribed to the activation of NF-*κ*B and PI3K/AKT signal pathways independent of TLR/IL-1R.^30, 31, 32^

Lnc-Myd88, as a novel lncRNA validated in our study, was first reported in human HCC. The particular aberrant expression of Lnc-Myd88 showing a positive regulation with Myd88 implied that there might be a regulation relationship between them. According to the analysis of Encode database, we found that Lnc-Myd88 was located upstream of the protein-coding gene Myd88, and presented a reverse transcription direction with Myd88. On account of the results of ChIP assays conducted above, Lnc-Myd88 showed a positive correlation with the enrichment of H3K27Ac in the promoter region of Myd88, indicating that Lnc-Myd88 might regulate Myd88 expression through histone modifications. And the following ChIP sequence experiments confirmed our hypothesis that Lnc-Myd88 enhanced Myd88 expression through increasing the enrichment of H3K27Ac in the promoter region and then activated the NF-*κ*B and PI3K/AKT signal pathways.

In conclusion, our study identified a novel Lnc-Myd88 which has positive relationship with neighbor tumor promoter Myd88 in human HCC. Lnc-Myd88 might increase Myd88 expression through enhanced H3K27Ac in the promoter of Myd88 gene. Aberrant expression of Lnc-Myd88 increased Myd88 levels, resulting in the activation of both NF-*κ*B and PI3K/AKT signal pathways and then promoting the proliferation and metastasis of HCC both *in vitro* and *in vivo*. Since enhanced Lnc-Myd88 expression indicated more aggressive tumors and worse clinical outcome in HCC, we proposed that Lnc-Myd88 might be a novel index for clinical diagnosis, and might serve as a potential therapeutic target for HCC. Collectively, all our findings are beneficial to explore the mechanism of lncRNAs in HCC carcinogenesis and progression, which can contribute to explore novel prognosis and potential therapeutic markers of HCC.

## Methods and materials

### Patient samples and cell lines

Total 110 paired HCC fresh tissues consist of tumors and adjacent normal samples were obtained from patients who underwent liver resection at the Liver Transplantation Center in The First Affiliate Hospital of Nanjing Medical University between October 2012 and November 2013. Approved by our Institutional Ethics Committee, all patients in our study offered their informed consent to take part in our study prior to surgery. All fresh tissues were collected and frozen in liquid nitrogen within 10 min. The diagnosis of all patients were histopathologically confirmed and the clinical characteristics of all patients are summarized in Table 1. The HepG2, SNU423, SMMC-7721, Hep3B, 97H, 97 L, Huh7 human hepatoma cell lines and the human normal L02 cell line used in this study were obtained from KeyGen (Nanjing KeyGen Biotech Co., Ltd, Jiangsu, China). All of the cells were cultured in DMEM medium (Gibco, New York, CA, USA) pre-treated with 10% fetal bovine serum, 80 U/ml of penicillin sodium at 37 °C in humidified air containing 5% carbon dioxide.

### Quantitative real-time PCR

Total RNAs of fresh tissue samples and cells were extracted with TRIzol reagent according to the manufacturer’s instructions (IInvitrogen, Carlsbad, CA, USA). The qRT-PCR was conducted to evaluate the expression level of Lnc-Myd88 and mRNAs of all relevant genes. To extract RNAs from cytoplasma and cytonucleus, we used the SurePrep Nuclear or Cytoplasmic RNA Purification Kit of Thermo Fisher Scientific (Rochester, Waltham, MA, USA). GAPDH were used as the internal control.

### Ectopic expression and gene silencing of Lnc-Myd88, gene silencing of Myd88

To overexpress Lnc-Myd88, full-length of Lnc-Myd88 was subcloned into the lentivirus vector GV367 (Gene, Shanghai, China). The shRNA sequence targeting Lnc-Myd88 was cloned into the lentivirus vector pLL3.7 (Gene) to knock down Lnc-Myd88 expression, and the negative control hairpin shRNA with no sequence homology to human genes was provided by the same manufacturer. Lentiviral plasmid vectors encoding short hairpin RNAs (shRNAs) targeting Myd88 or scramble shRNA were generated and designated as sh-Myd88 and sh-NC, respectively. All the vectors were labeled with enhanced green fluorescence protein (EGFP). Transfection of cells was conducted according to the manufacturer instructions, and the efficiency of transfection was validated by qRT-PCR, and then the cells were subjected to RNA extraction or functional assays.

### Cell proliferation and invasion assay

The proliferation ability of HCC cells was tested by Clone Formation assay, Cell Counting Kit-8 (Dojindo Laboratories, Kumamoto, Japan), EDU (5-ethynyl-2′-deoxyuridine) immunofluorescence staining assay (Milipore, Billerica, MA, USA) according to the manufacturer’s instructions. Cell migration and invasion assays were conducted using transwell chamber (8*μ*m pore size; Millipore). In the migration assays, cells were cultured in the upper chamber with serum-free medium. However, in the invasion assays, the lower chamber was added in media supplemented with 10% FBS. After 48 h, cells that had migrated or invaded through the membrane were fixed with methanol, stained with crystal violet and counted.

### Flow cytometry analysis of cell cycle and apoptosis

For cell-cycle analysis, Lv-NC-SMMC-7721, Lv-Lnc-Myd88-SMMC-7721, sh-NC-Huh7 and sh-Lnc-Myd88-Huh7 cells were subjected to serum starvation to induce cell-cycle synchronization. The cells at the logarithm growth period were harvested and fixed in 70% ethanol for a night at −20 °C. The next day, the cells were washed and incubated in propidium iodide and analyzed by flow cytometry. For apoptosis analysis, cells were cultured with complete medium with 0.5 mM peroxide overnight, and Annexin V-APC/7-AAD staining also was performed by using flow cytometry according to the manufacturer (Roche, Basel, Switzerland).

### *In vivo* experiments

BAB/c nude mice, 6 weeks of age or older, were purchased from the animal center of Nanjing University (Nanjing, Jiangsu, China), raised and permitted by the Nanjing medical University animal studies committee. In the subcutaneous transplantation model, five mice were implanted with Lv-Lnc-Myd88-SMMC-7721 cells (1 × 10^7^) in the right groin and Lv-NC SMMC-7721 cells (1 × 10^7^) in the left groin. We calculated the volume of tumors every 5 days after transplantation and killed them 30 days after implantation. For the tail vein xenograft model, eight mice in each group were injected with cells (1 × 10^7^ suspended in 200 *μ*l PBS) through the tail vein and killed 5 weeks later. One group was sh-Lnc-Myd88-Huh7 cells and the other was sh-NC-Huh7 cells which were all labeled with EGFP. Tumors of lungs were visualized by fluorescence using a 470-nm light source (Lightools Research, Encinitas, CA, USA).

### Immunohistochemical assay

The tissue samples were fixed in 4% paraformaldehyde at 4 °C and sectioned into slices. After deparaffinage and rehydration, the sections were put into pressure cooker for 5 min to restore the antigen by using the citrate method. H_2_O_2_ suppresses endogenous peroxidase activity to reduce background. Blocked in normal goat serum with 5% BSA in TBS for 1 h at room temperature was also needed. The sections were incubated with primary antibody (1:400 dilutions) overnight at 4 °C and then washed in PBS for three times. After incubated with secondary antibodies, sections were subjected to DAB reaction. Photograph of the sections by using a digitalized microscope camera (Nikon, Tokyo, Japan).

### Chromatin immunoprecipitation

ChIP was performed by using the ChIP assay kit according to the manufacturer’s protocol (17-610; Millipore). 1x10^7^–5x10^7^ cells were collected. Formaldehyde is used to crosslink the proteins to the DNA for 20–30 min. Then sonicate lysate to shear DNA to a fragment size of 200–1000 bp. After determination of DNA concentration and fragment size, we add the primary antibody, anti-H3K27m3, anti-H3K27ac, anti-H3K4m3 and IgG, and protein A/G beads into the samples and incubated overnight at 4 °C. The crosslinking was reversed by incubation at 65 °C for 4 h. The DNA was recovered by phenol/chloroform extraction. The primers were used to detect the human Myd88 promoter region by PCR.

### Western blotting

To get protein, tissue samples and cultured cells were dissolved by RIPA regent plus phenylmethanesulfonylfluoride (Beyotime, Nantong, China). Consistently, 30 mg of the protein was loaded each lane, fractionated by SDS PAGE, transferred onto a PVDF membrane. And then the membrane was incubated at 4 °C overnight with human-specific antibody of Myd88 (Abcam, London, UK), p-NF-*κ*B and NF-*κ*B (CST, Boston, MA, USA), p-AKT/AKT (Abcam, London, UK), GAPDH (CST). The results were visualized by a chemiluminescent detection system (Pierce ECL substrate western blot detection system; Thermo Scientific, Waltham, MA, USA) and exposure to autoradiography film.

### Statistical analysis

All experimental assays were repeated independently in triplicate. Data were expressed as mean±S.E.M. Two-tailed Student's *t-*test was used to assess the statistical differences between groups. All statistical data were carried out using Statistical Program for Social Sciences 19.0 software (SPSS, Palo Alto, CA, USA) and presented with Graphpad prism 5.0 (GraphPad Software, La Jolla, CA, USA). *P*-value less than 0.05 was considered as significant.

## Publisher’s Note

Springer Nature remains neutral with regard to jurisdictional claims in published maps and institutional affiliations.

## Figures and Tables

**Table 1:** Correlation between Lnc-Myd88 expression and clinicopathological characteristics of HCC patients (*n*=110)

**Characteristics**	**All patients**	**Lnc-Myd88 low expression (<median**^a^)	**Lnc-Myd88 high expression (≥median**)	**p Chi-squared test *P*-value**
*n*	110	55	55	
				
*Age (years)*				
<60	77	36	41	0.298
≥60	33	19	14	
				
*Gender*				
Male	89	46	43	0.467
Female	21	9	12	
				
*HbeAg*				
Negative	39	23	16	0.163
Positive	71	32	39	
				
*Cirrhosis*				
Absent	32	18	14	0.401
Present	78	37	41	
				
*AFP (ng/ml)*				
≤13.6	32	17	15	0.675
>13.6	78	38	40	
				
*Tumor size (cm)*				
≤5	36	25	11	0.004*
>5	74	30	44	
				
*Tumor number*				
Single	88	43	45	0.634
Multiple	22	12	10	
				
*Metastasis*				
Yes	23	6	17	0.010*
No	87	49	38	
				
*Edmondson grade*				
I+II	79	46	33	0.006*
III+IV	31	9	22	

*P-value <0.05

a The median expression level of Lnc-Myd88 was used as the cutoff.
