# Correction: Long non-coding RNA Myd88 promotes growth and metastasis in hepatocellular carcinoma via regulating Myd88 expression through H3K27 modification

**DOI:** 10.1038/s41419-024-07101-x

**Published:** 2024-12-03

**Authors:** Xiaoliang Xu, Yin Yin, Junwei Tang, Yu Xie, Zhuo Han, Xudong Zhang, Qiaoyu Liu, Xihu Qin, Xinli Huang, Beicheng Sun

**Affiliations:** 1 Liver Transplantation Center of the First Affiliated Hospital and State Key Laboratory of Reproductive Medicine, Nanjing Medical University, Nanjing, Jiangsu Province PR China; 2 Department of General Surgery, Huai’an First People’s Hospital, Nanjing Medical University, Huai’an, Jiangsu Province PR China; 3 The Affiliated Changzhou NO.2 People’s Hospital of Nanjing Medical University, Changzhou, Jiangsu Province PR China

Correction to: *Cell Death & Disease* 10.1038/cddis.2017.519, published online 12 October 2017

The original version of this article unfortunately contained a mistake in Figure 3c.

In this article, we find that there is a misplaced subfigure in Figure 3c. In the transwell assays of SMMC-7721 Lv-NC cells, we incorrectly placed one result originally belonging to the migration assay in the results of the invasion assay. After double checking the original data, it appeared that we misused the picture in the control group of invasion (SMMC-7721 Lv-NC) (Figure 3c) due to misnaming. We sincerely apologize for this mistake and confirm that this error do not affect the conclusions of the article. And we are so sorry for the inconvenience caused by the mistake for readers.

The corrected figure can be found below. The authors apologize for the mistake.
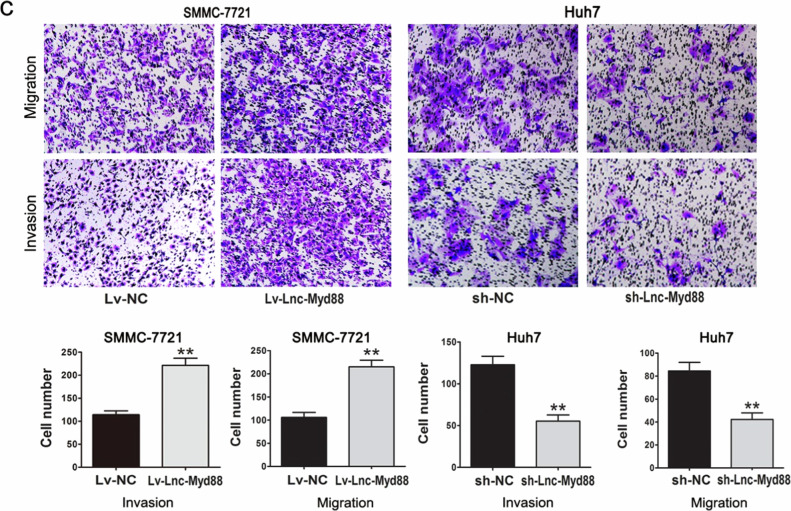


## Supplementary information

original data for CDD

