# Supplementary material for: Long non-coding RNA Myd88 promotes growth and metastasis in hepatocellular carcinoma via regulating Myd88 expression through H3K27 modification
Source: Cell Death Dis. 2017 Oct 12;8(10):e3124–. doi: 10.1038/cddis.2017.519 (PMC5682683; doi:10.1038/cddis.2017.519)
Supplement: Supplementary Information [file cddis2017519x1.pdf]

Supplementary Information for:

Long non-coding RNA Myd88 promotes growth and metastasis in hepatocellular carcinoma via regulating Myd88 expression through H3K27 modification

Xiaoliang Xu[1, #], Yin Yin[1, #], Junwei Tang[1, #], Yu Xie[3], Zhuo Han[1], Xudong Zhang[2], Qiaoyu Liu[1], Xihu Qin[2, *], Xinli Huang[1, *], Beicheng Sun[1, *]

1 Liver Transplantation Center of the First Affiliated Hospital and State Key Laboratory of Reproductive Medicine, Nanjing Medical University, Nanjing, Jiangsu Province, P.R. China.

2 The Affiliated Changzhou NO.2 People's Hospital of Nanjing Medical University, Changzhou, Jiangsu Province, P.R. China.

3 Department of General Surgery, Huai'an First People's Hospital, Nanjing Medical University, Huai'an, Jiangsu Province, P.R. China.

#The authors contributed equally to this work.

*Corresponding authors: Dr. Beicheng Sun, Liver Transplantation Center of the First Affiliated Hospital and State Key Laboratory of Reproductive Medicine, Nanjing Medical University, Nanjing, Jiangsu Province, P.R.China.. Phone: 86-25-68136746, Fax: 86-25-86560946, E-mail: sunbc@njmu.edu.cn. Dr. Xinli Huang, Liver Transplantation Center of the First Affiliated Hospital and State Key Laboratory of Reproductive Medicine, Nanjing Medical University, Nanjing, Jiangsu Province, P.R.China. E-mail: huangxinli@nimu.edu.cn. Dr. Xihu Qin, The Affiliated Changzhou NO.2 People's Hospital of Nanjing Medical University, Changzhou, Jiangsu Province, P.R. China. E-Mail: qinxihu@126.com.

Contents:

1. Supplementary figure legend

1. Supplementary figure legend

Supplementary Figure 1: Non-coding RNA validation and the expression of Lnc-Myd88 in cell lines.

(A) Non-coding RNA validation by bioinformatics prediction, comparing with ANRIL, a well-known non-coding RNA, Lnc-Myd88 was more likely to be a non-coding RNA. (B) Subcellular localization investigation indicated that the transcript for Lnc-Myd88 was located mainly in the nucleus of SMMC-7721 and Huh7 cell lines, according to the results of RT-PCR amplified with separated cytoplasm RNA and nucleus RNA. HPRT was used as the control for cytoplasmic expression and U2 for cytonuclear expression. (C) Different expression level of Lnc-Myd88 in HCC cells. (D) The levels of Myd88 and GAPDH were examined by western blotting in SMMC-7721 and Huh7 cells treated with sh-Myd88.

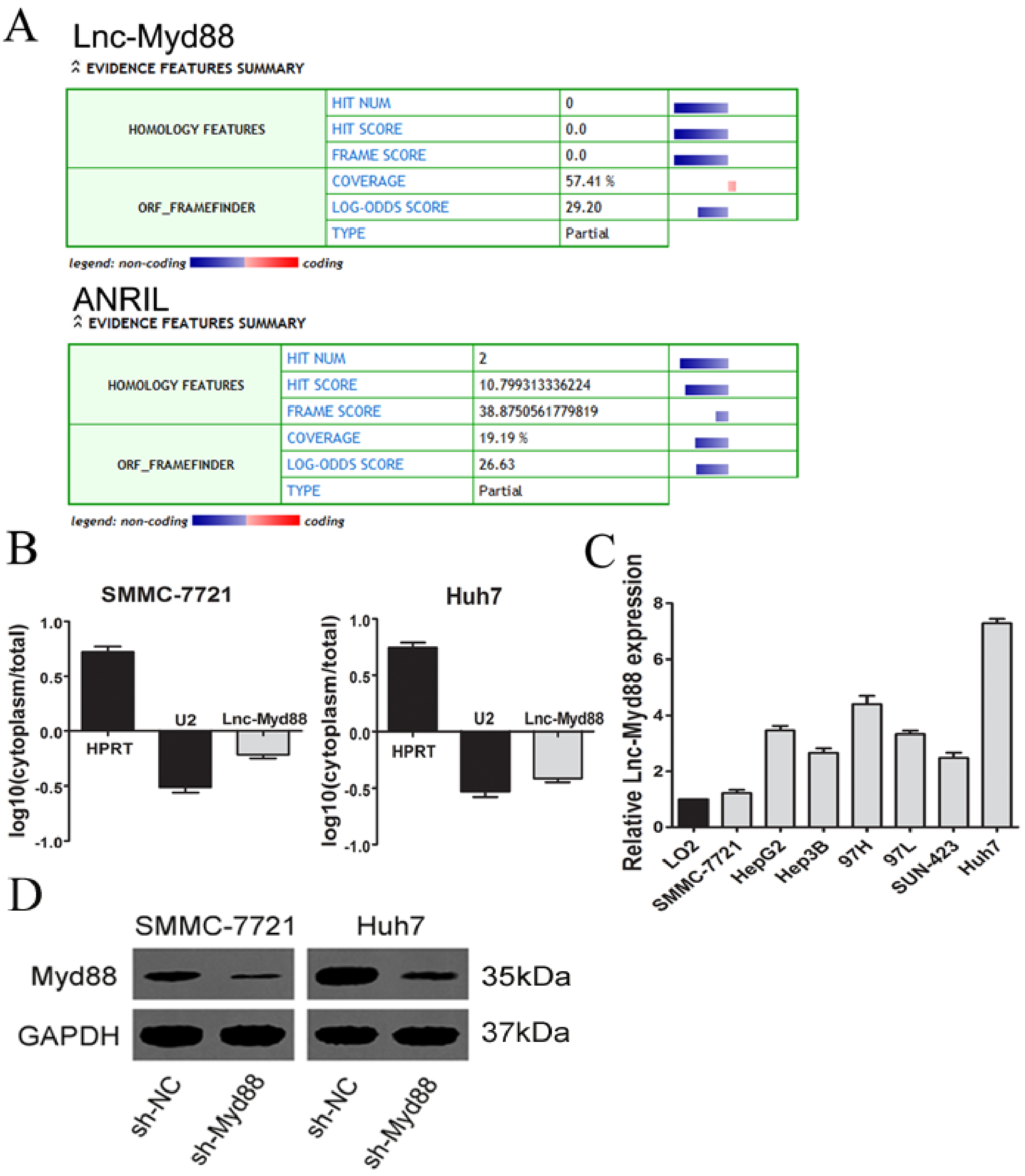
A
Lnc-Myd88
EVIDENCE FEATURES SUMMARY
HOMOLOGY FEATURES
HIT NUM 0
HIT SCORE 0.0
FRAME SCORE 0.0
ORF_FRAMEFINDER
COVERAGE 57.41 %
LOG-ODDS SCORE 29.20
TYPE Partial
legend: non-coding coding
ANRIL
EVIDENCE FEATURES SUMMARY
HOMOLOGY FEATURES
HIT NUM 2
HIT SCORE 10.799313336224
FRAME SCORE 38.8750561779819
ORF_FRAMEFINDER
COVERAGE 19.19 %
LOG-ODDS SCORE 26.63
TYPE Partial
legend: non-coding coding
B
SMMC-7721
Huh7
log10(cytoplasm/total)
1.0
0.5
0.0
-0.5
-1.0
HPRT
U2
Lnc-Myd88
C
Relative Lnc-Myd88 expression
8
6
4
2
0
LO2
SMMC-7721
HepG2
Hep3B
97H
97L
SUN-423
Huh7
D
SMMC-7721
Huh7
Myd88
GAPDH
35kDa
37kDa
sh-NC
sh-Myd88
sh-NC
sh-Myd88
