# Supplementary material for: Correction: Long non-coding RNA Myd88 promotes growth and metastasis in hepatocellular carcinoma via regulating Myd88 expression through H3K27 modification
Source: Cell Death Dis. 2024 Dec 3;15(12):876. doi: 10.1038/s41419-024-07101-x (PMC11615368; doi:10.1038/s41419-024-07101-x)
Supplement: Supplementary file 1 — original data for CDD [file 41419_2024_7101_MOESM1_ESM.pdf]

The original data of migration assay

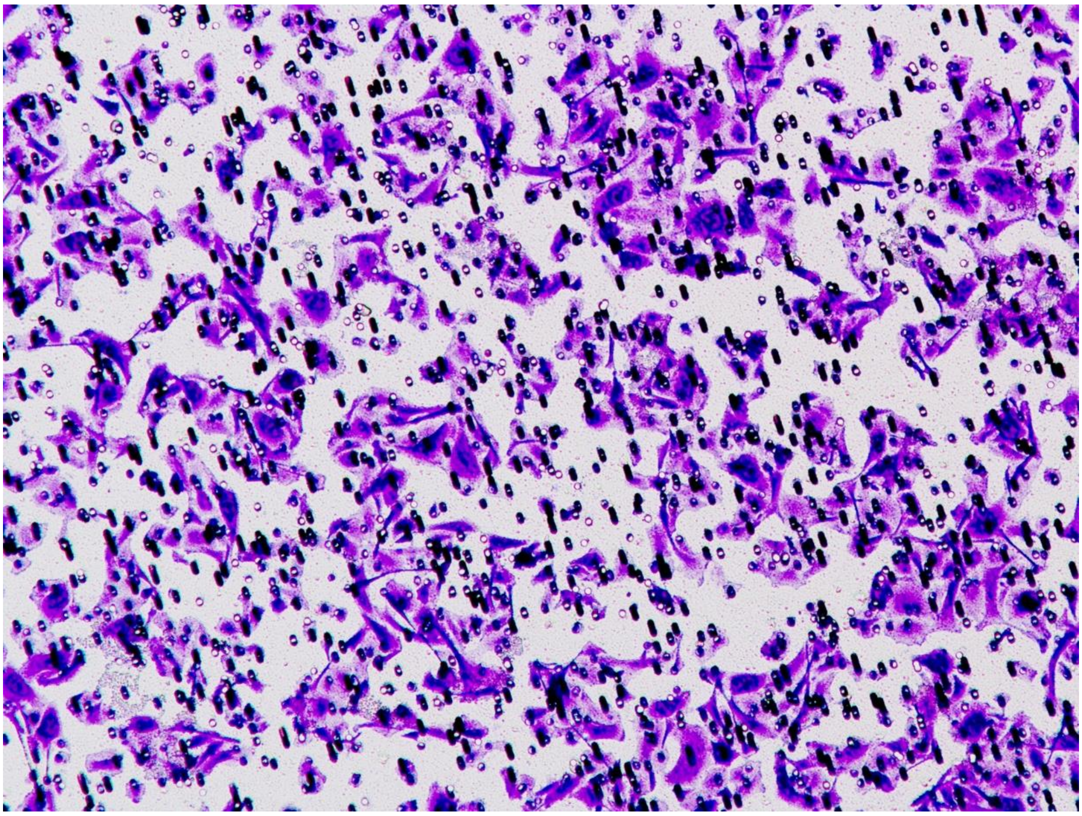

Picture 1: The first experiment of SMMC-7721 Lv-NC group

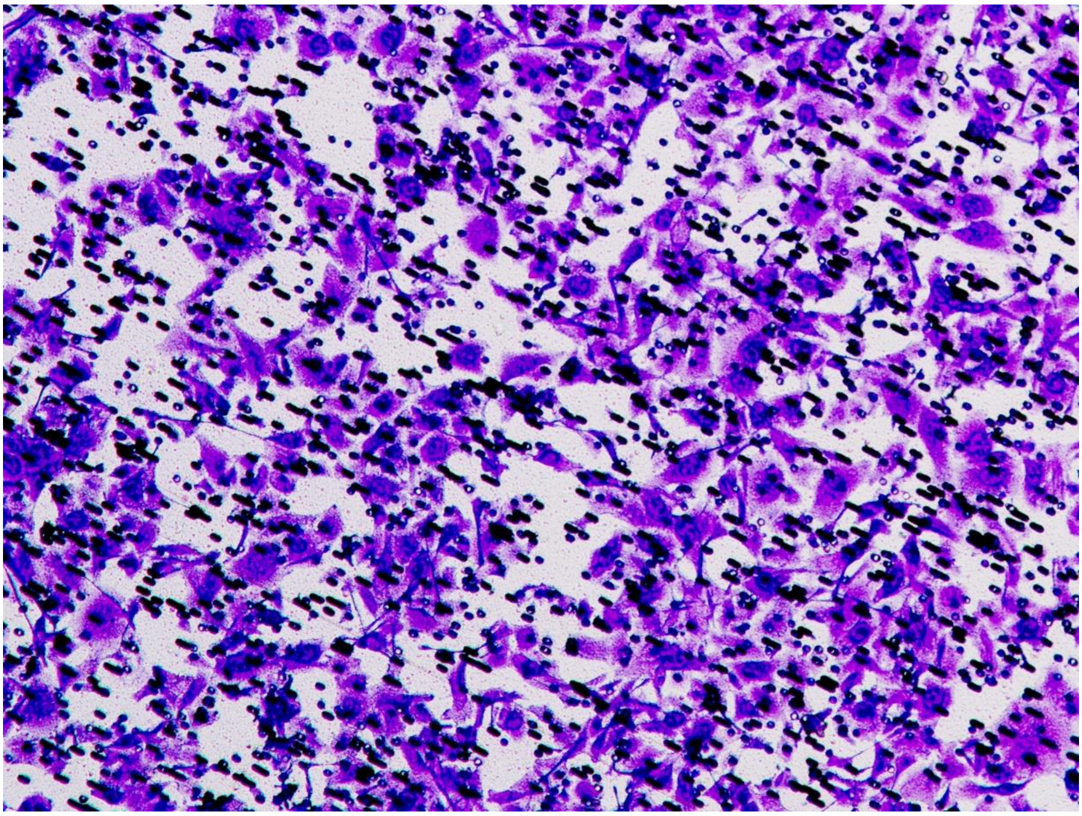

Picture 2: The first experiment of SMMC-7721 Lv-Lnc-Myd88 group

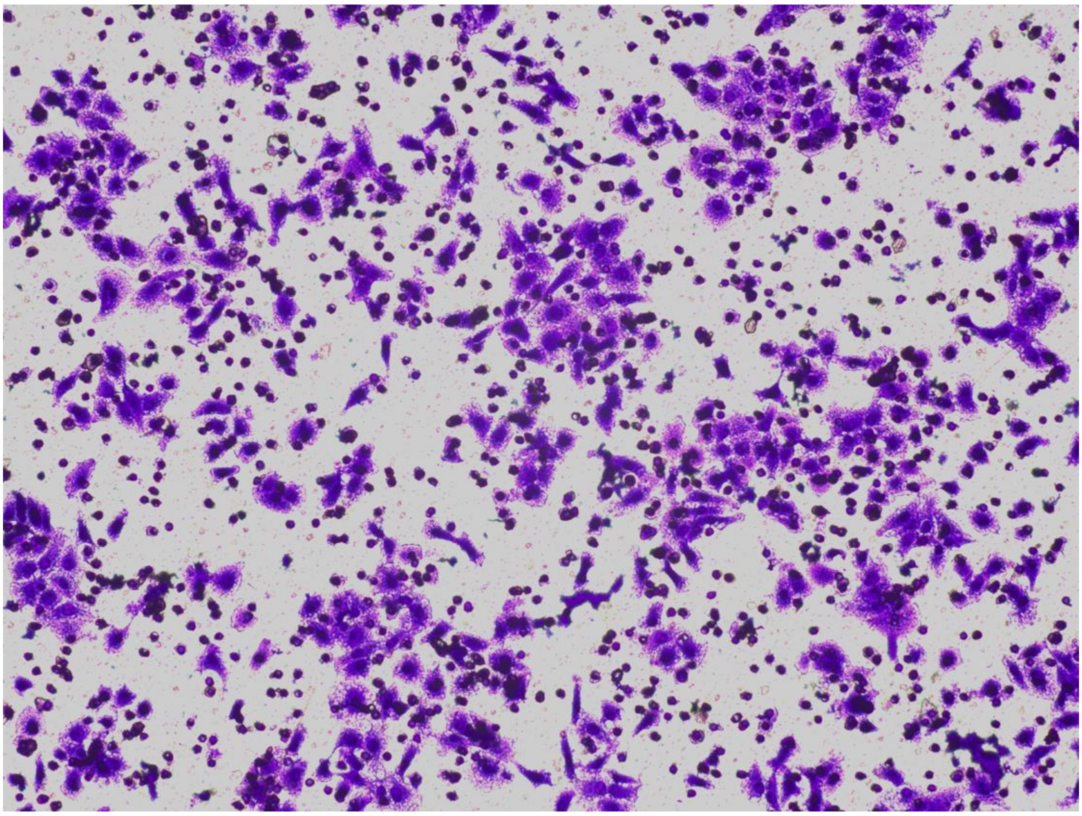

Picture 3: The second experiment of SMMC-7721 Lv-NC group

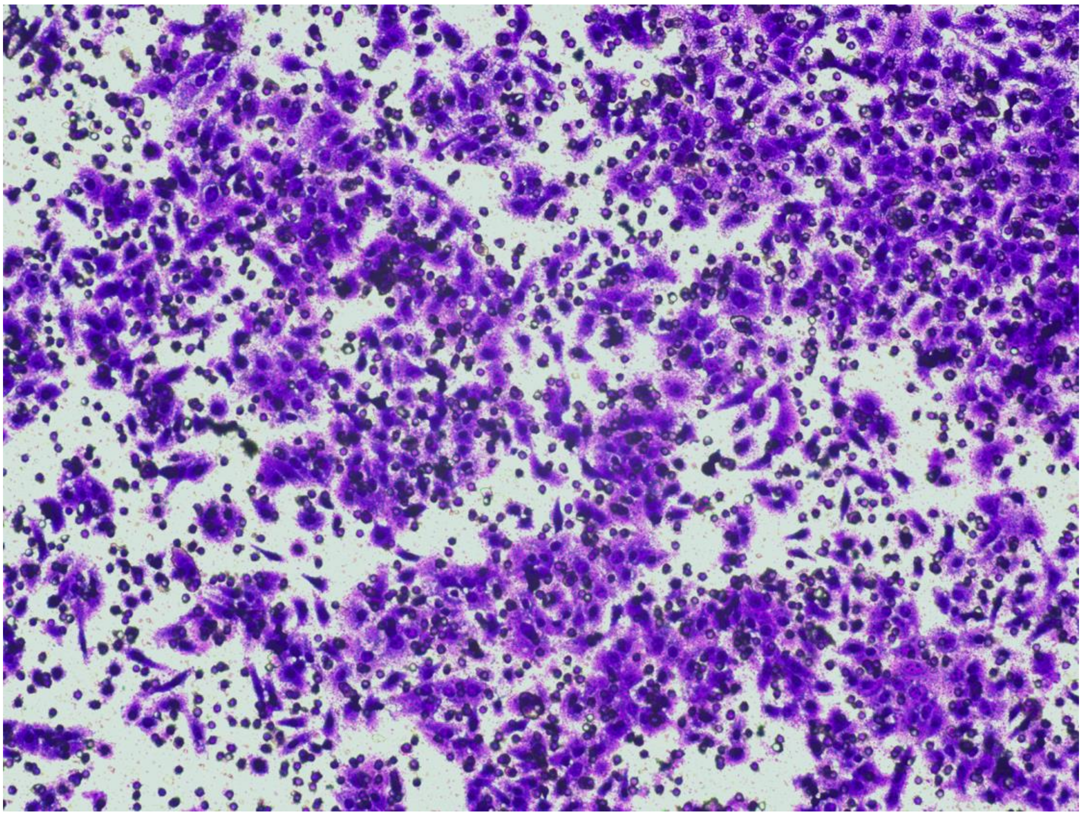

Picture 4: The second experiment of SMMC-7721 Lv-Lnc-Myd88 group

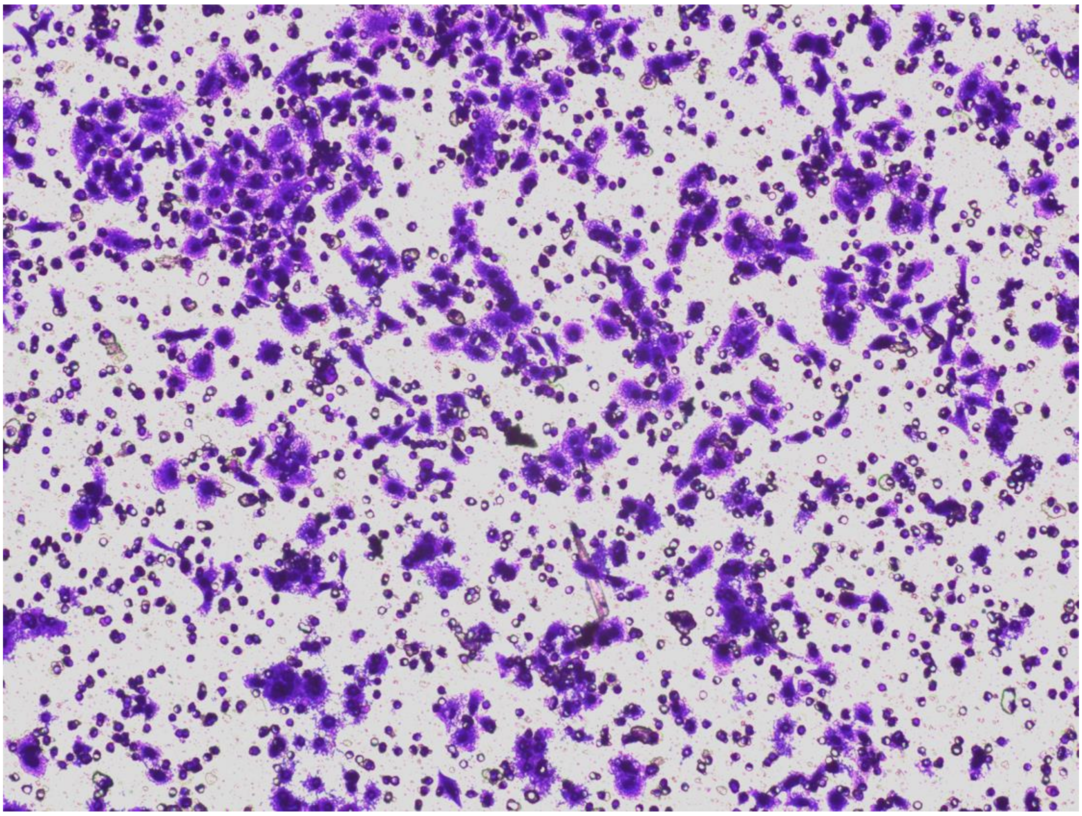

Picture 5: The third experiment of SMMC-7721 Lv-NC group

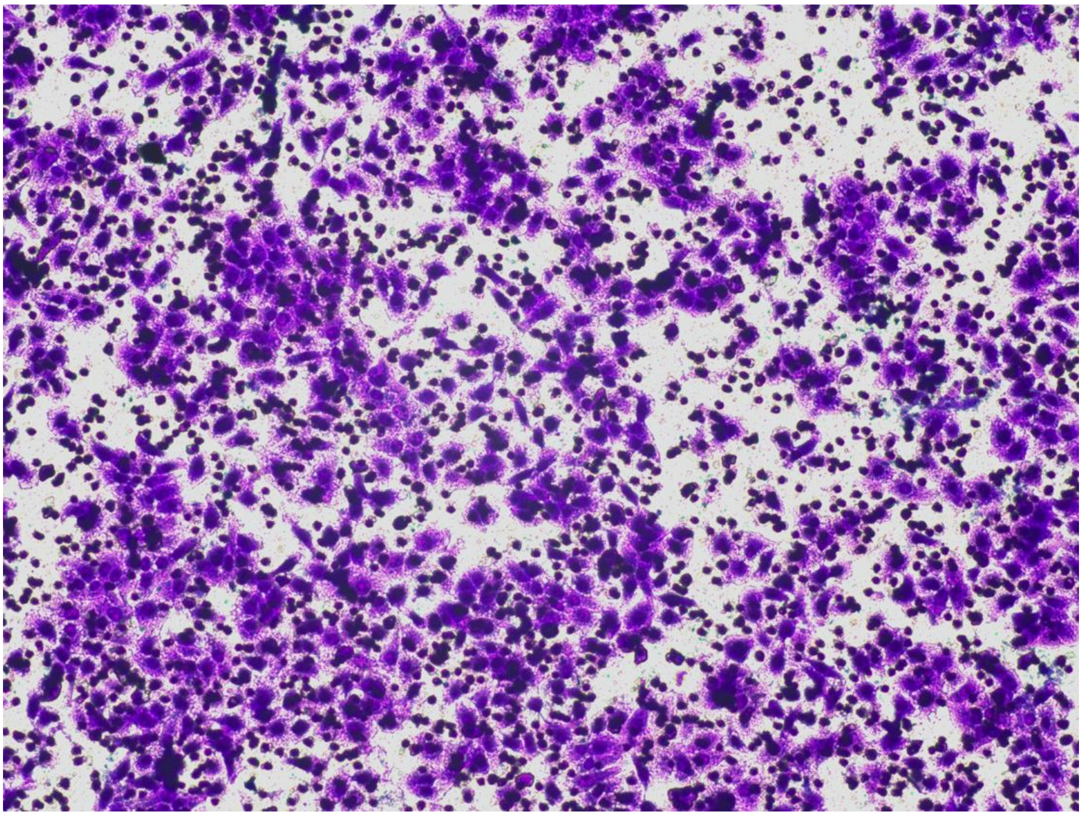

Picture 6: The third experiment of SMMC-7721 Lv-Lnc-Myd88 group

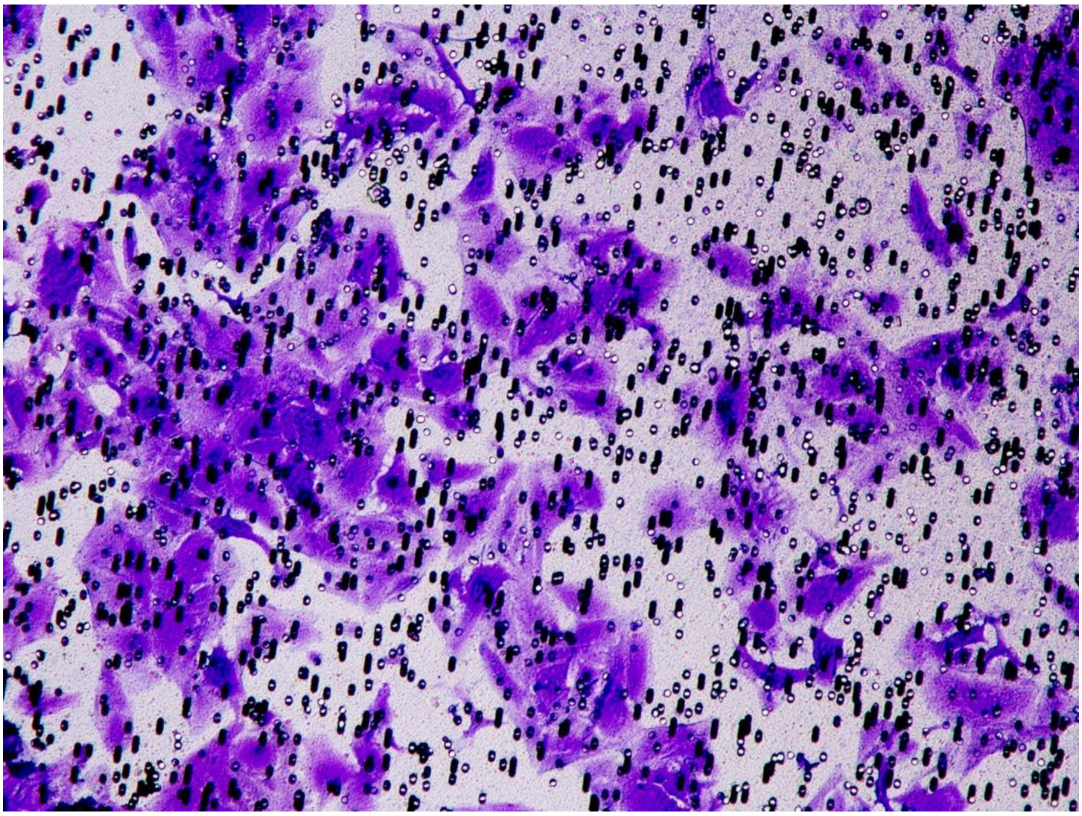
Picture 7: The first experiment of Huh7 sh-NC group

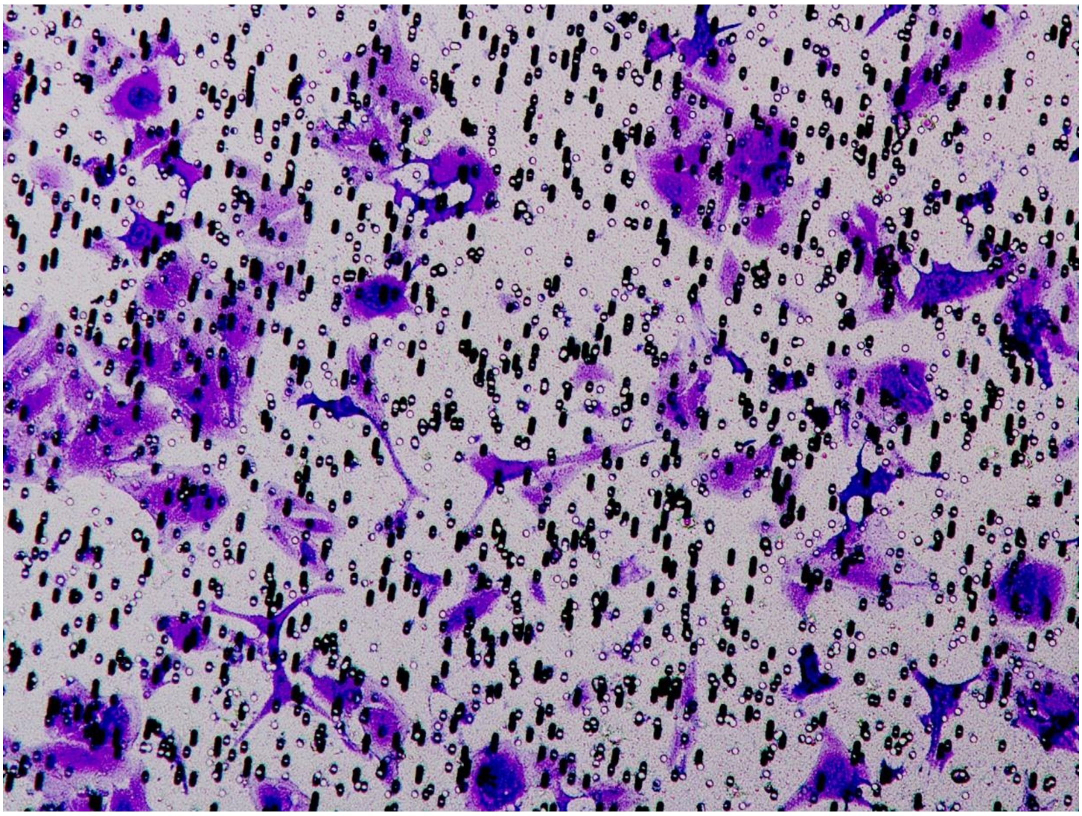
Picture 8: The first experiment of Huh7 sh-Lnc-Myd88 group

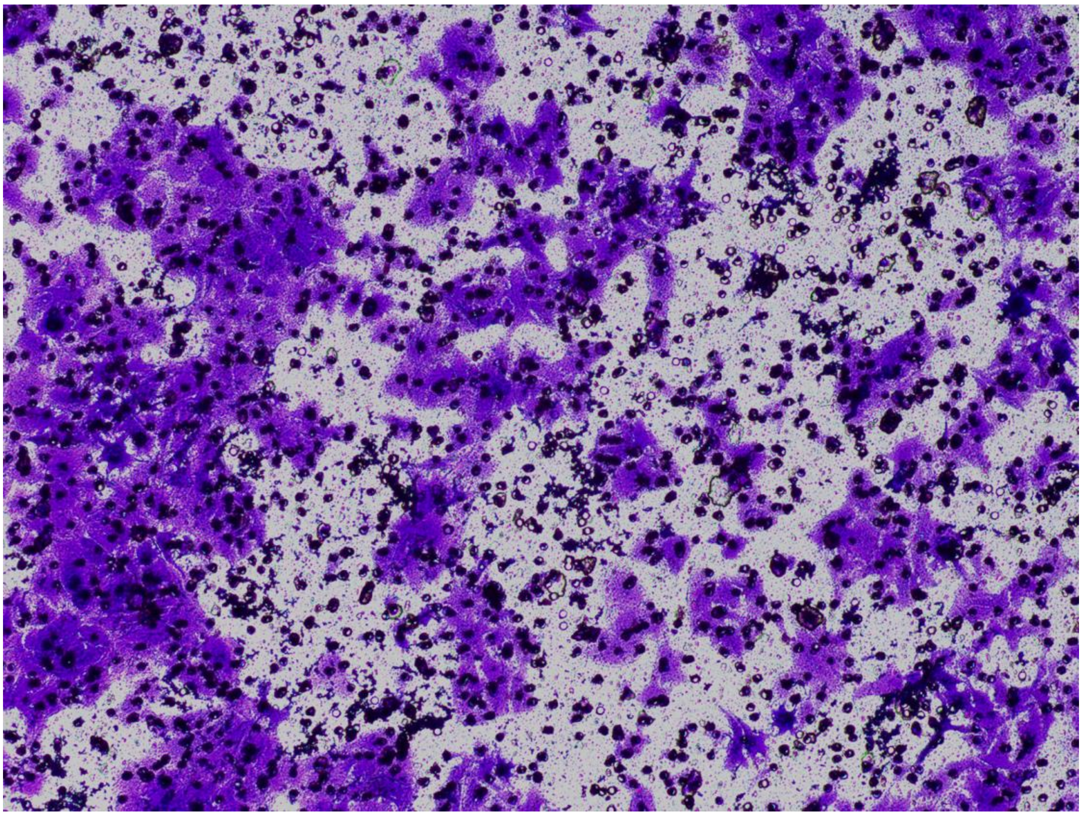

Picture 9: The second experiment of Huh7 sh-NC group

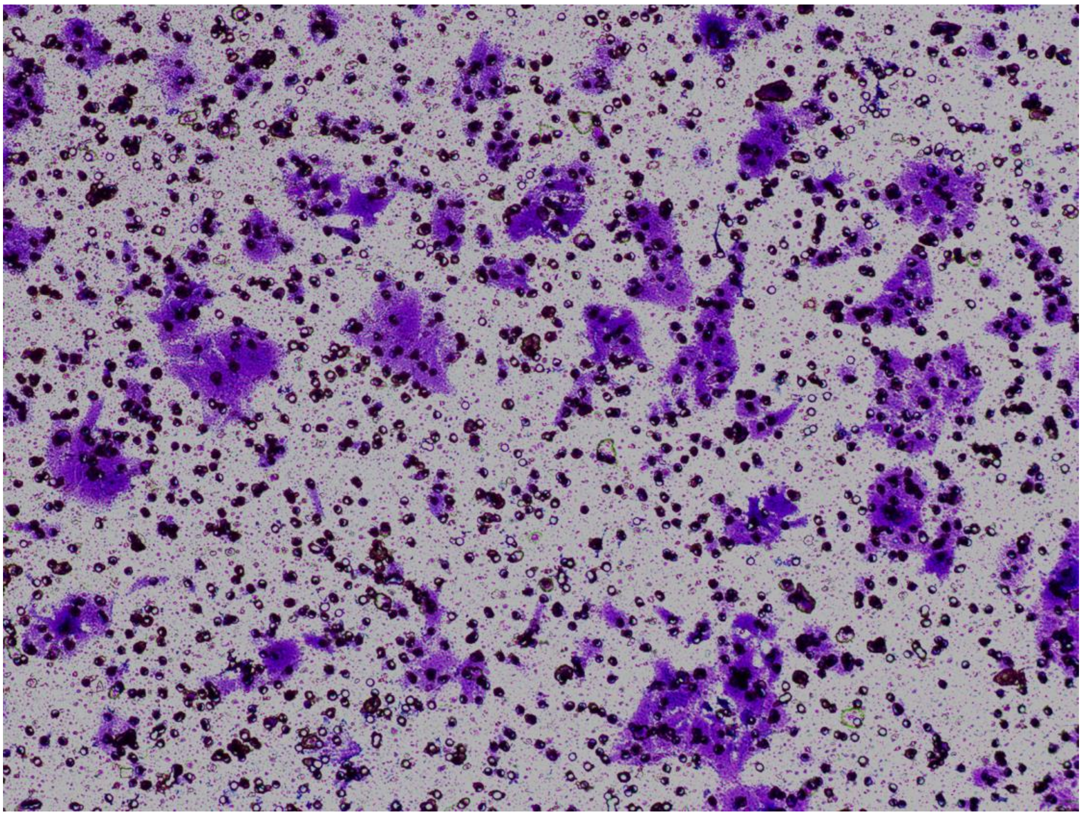

Picture 10: The second experiment of Huh7 sh-Lnc-Myd88 group

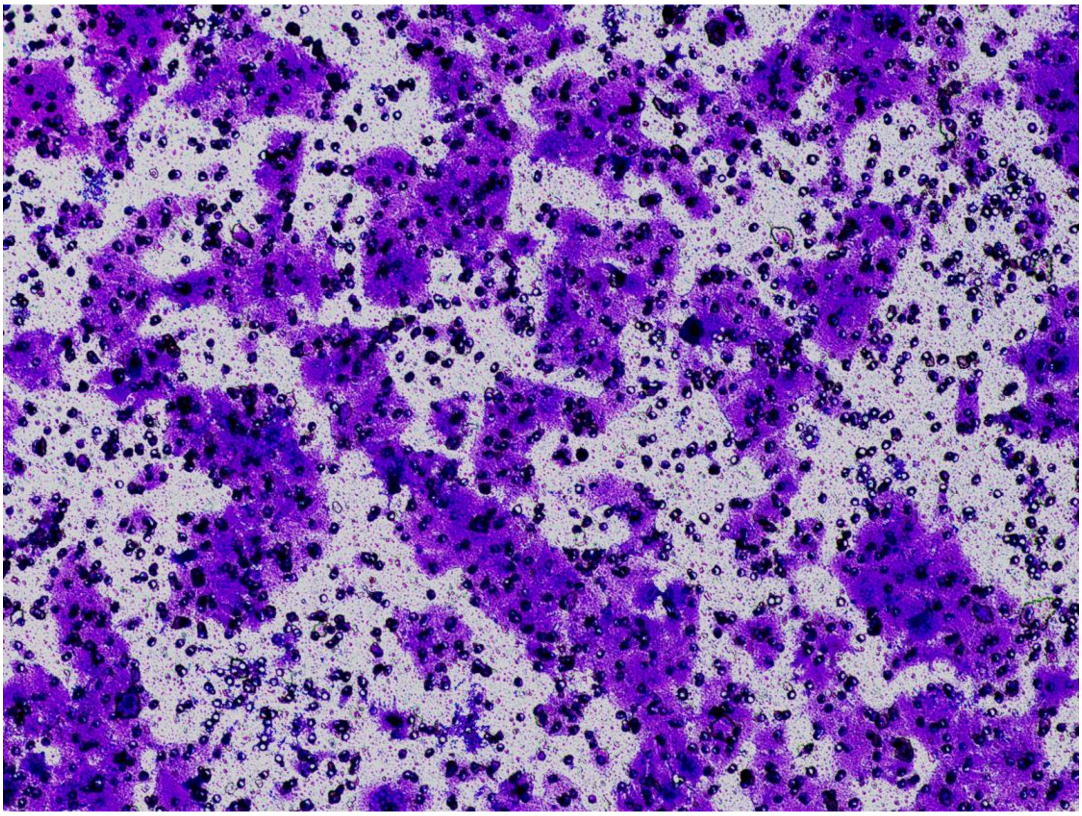

Picture 11: The third experiment of Huh7 sh-NC group

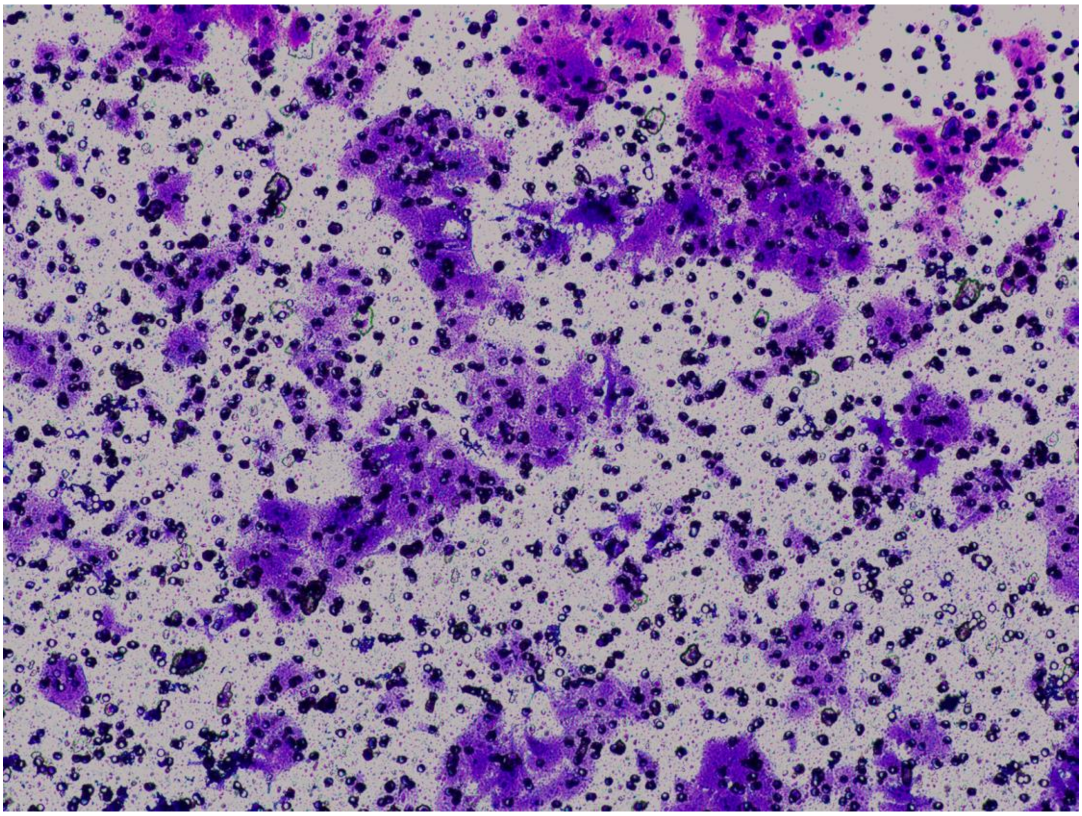

Picture 12: The third experiment of Huh7 sh-Lnc-Myd88 group

The original data of invasion assay

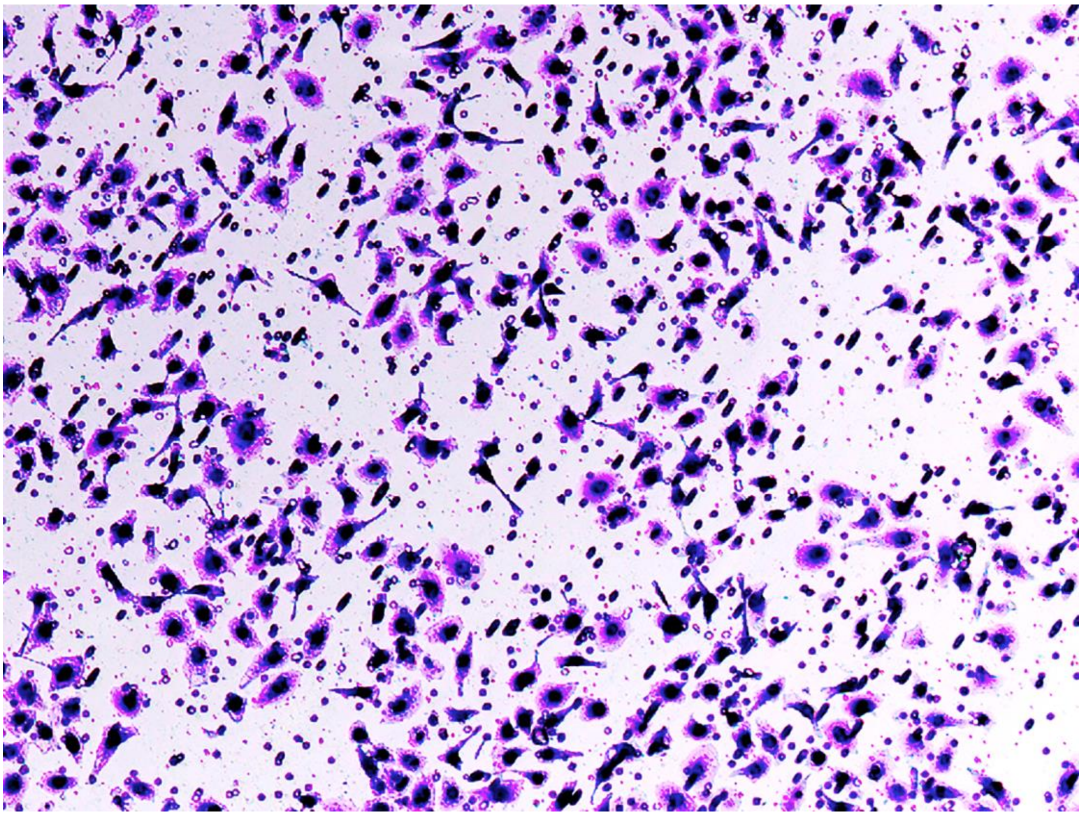

Picture 13: The first experiment of SMMC-7721 Lv-NC group

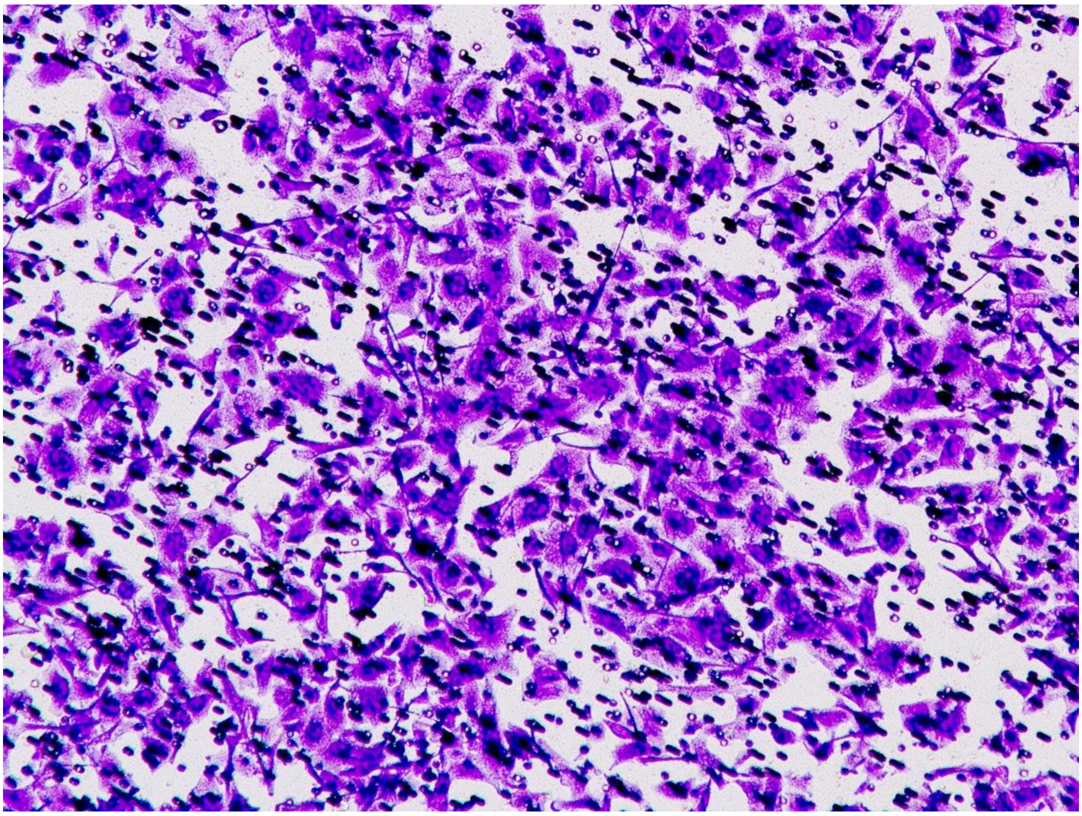

Picture 14: The first experiment of SMMC-7721 Lv-Lnc-Myd88 group

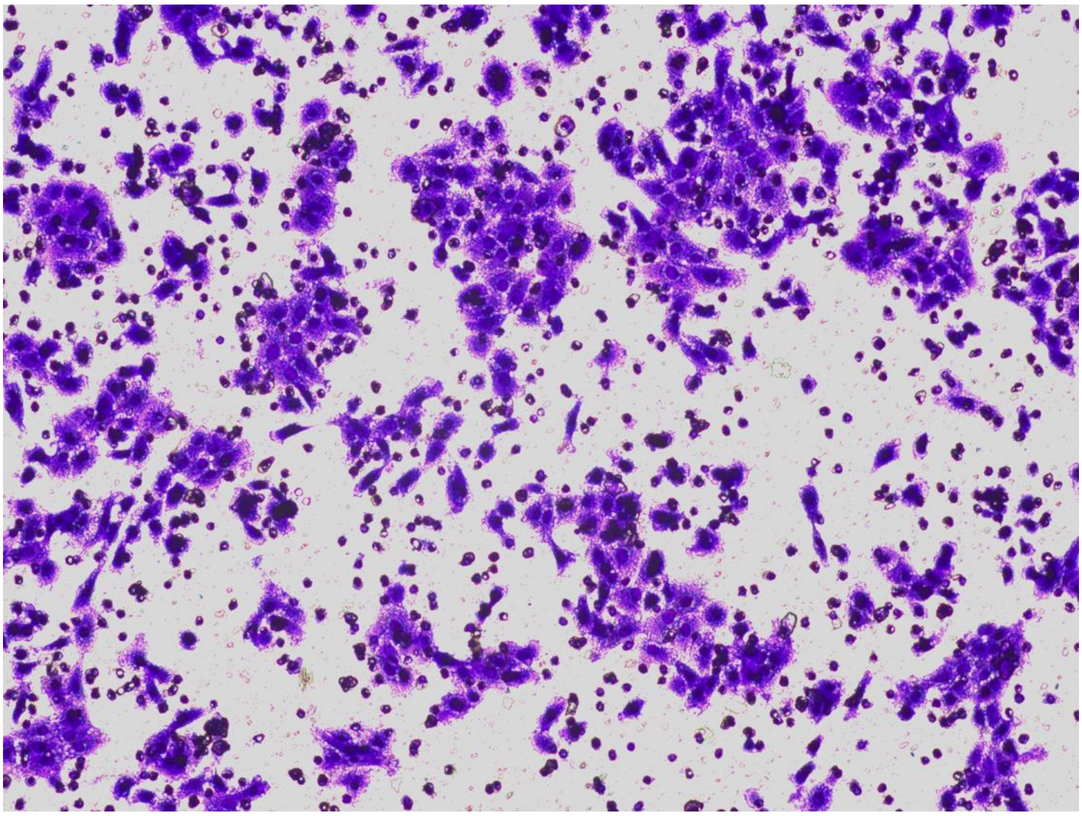

Picture 15: The second experiment of SMMC-7721 Lv-NC group

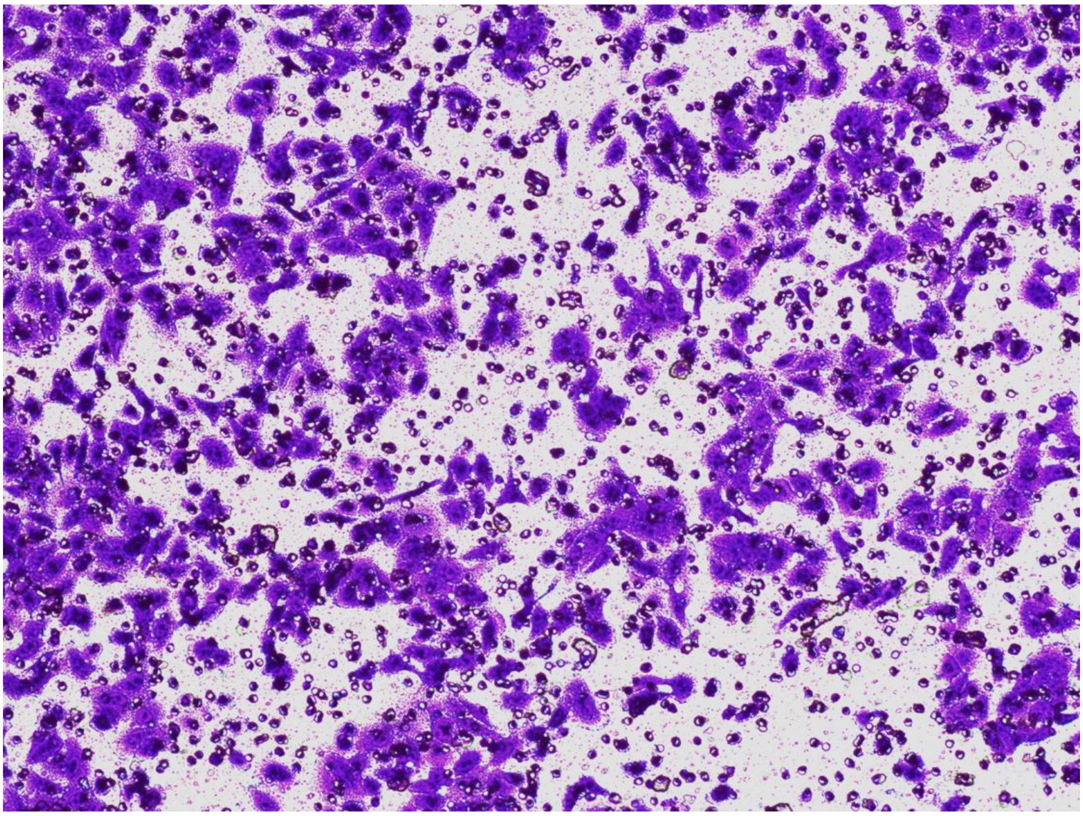

Picture 16: The second experiment of SMMC-7721 Lv-Lnc-Myd88 group

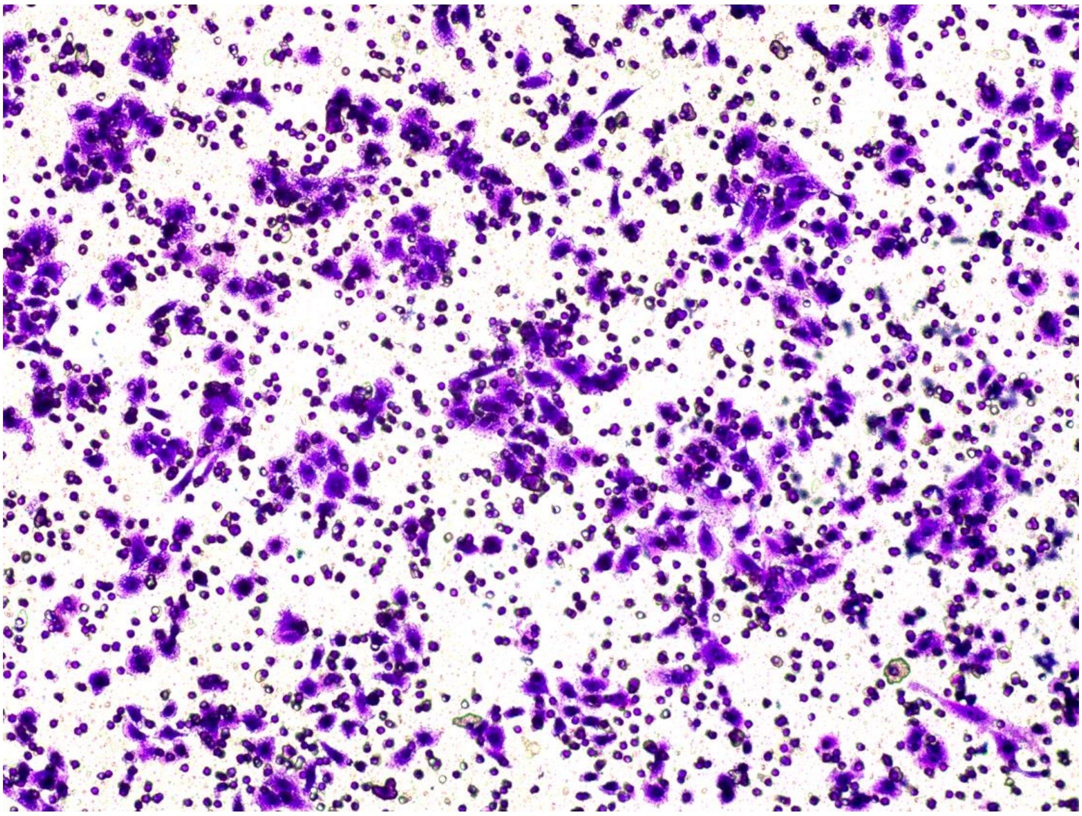

Picture 17: The third experiment of SMMC-7721 Lv-NC group

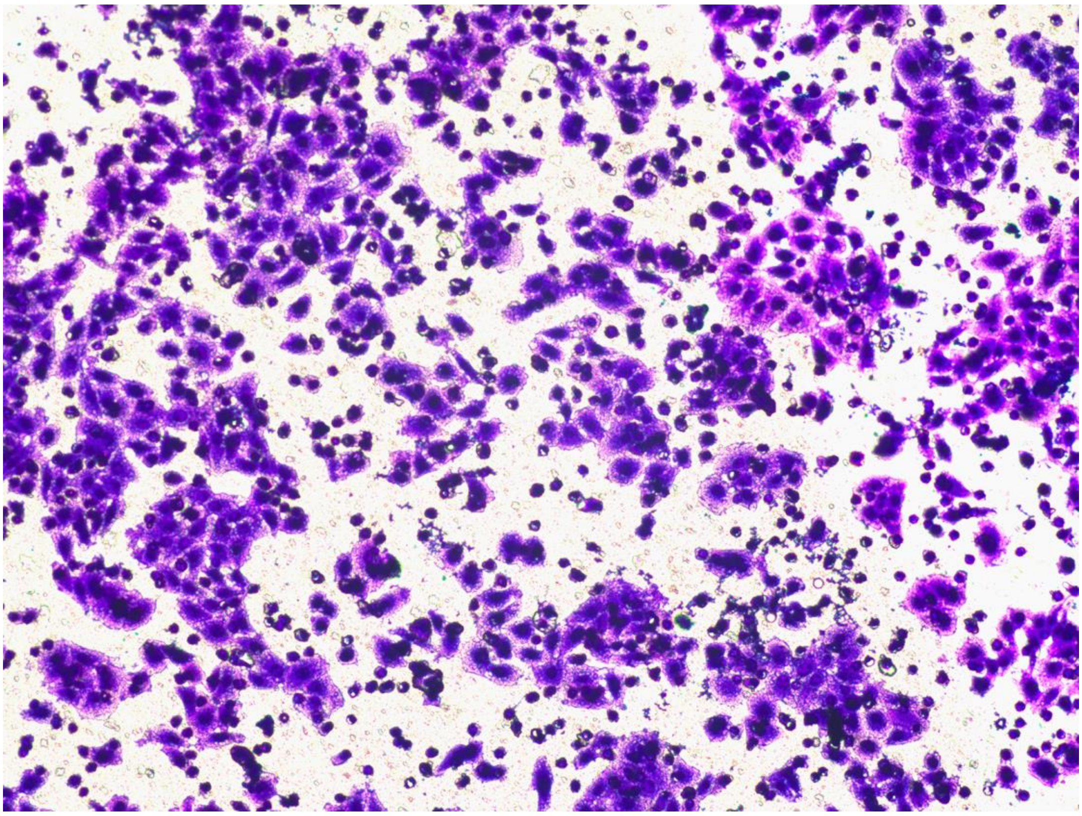

Picture 18: The third experiment of SMMC-7721 Lv-Lnc-Myd88 group

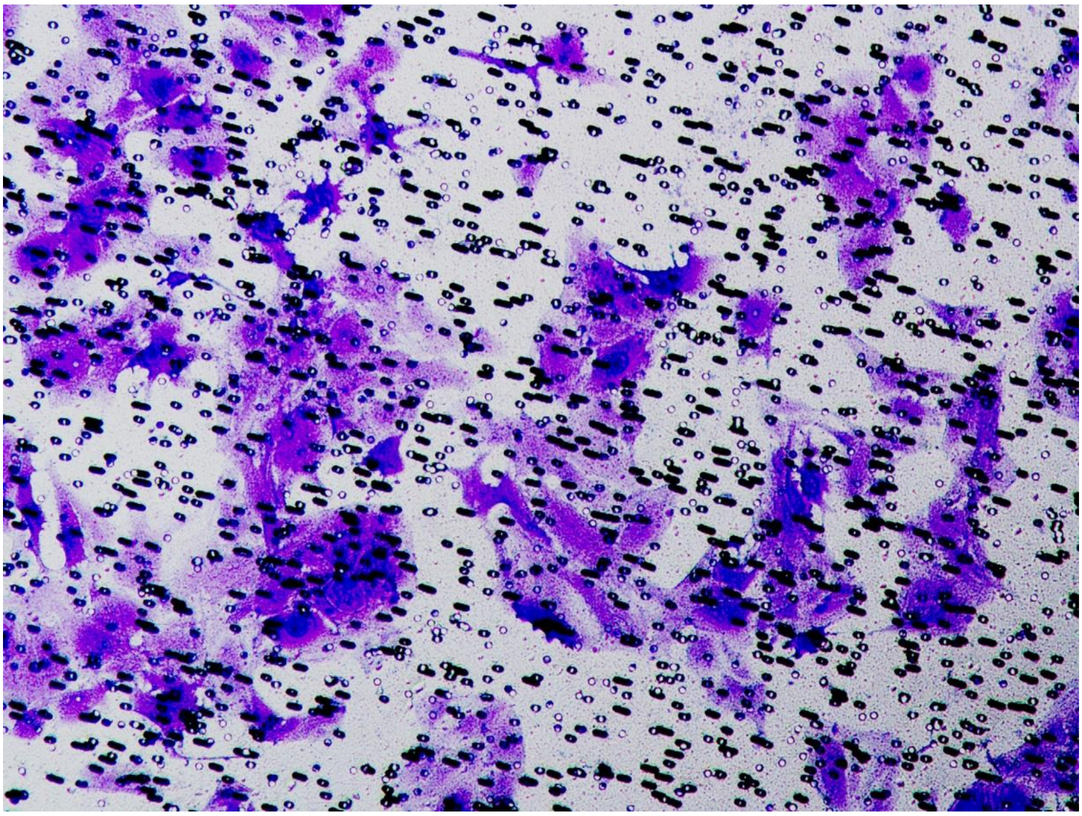

Picture 19: The first experiment of Huh7 sh-NC group

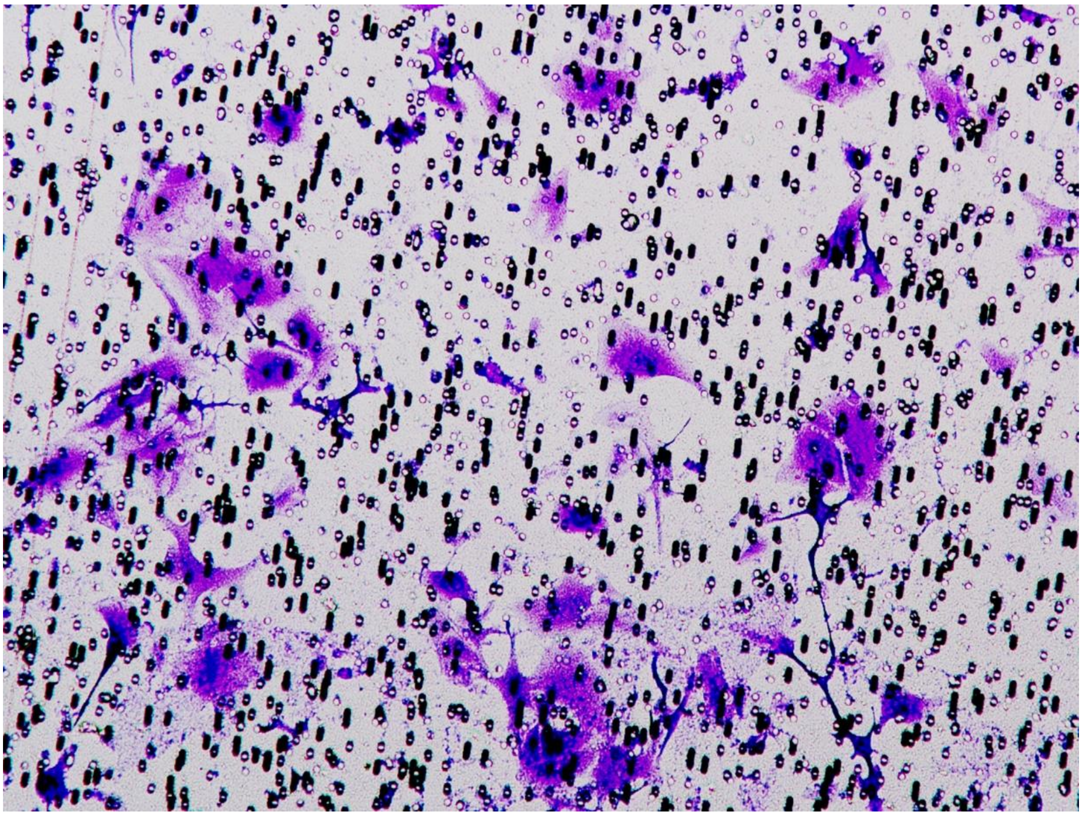

Picture 20: The first experiment of Huh7 sh-Lnc-Myd88 group

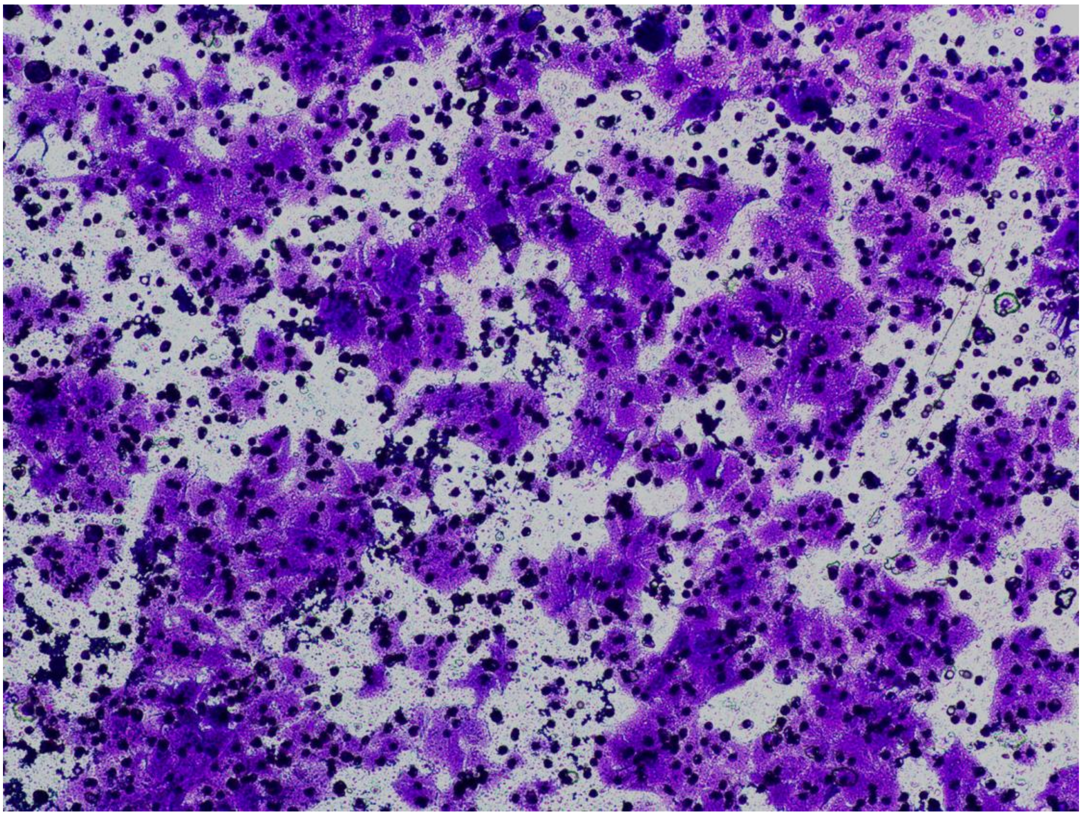

Picture 21: The second experiment of Huh7 sh-NC group

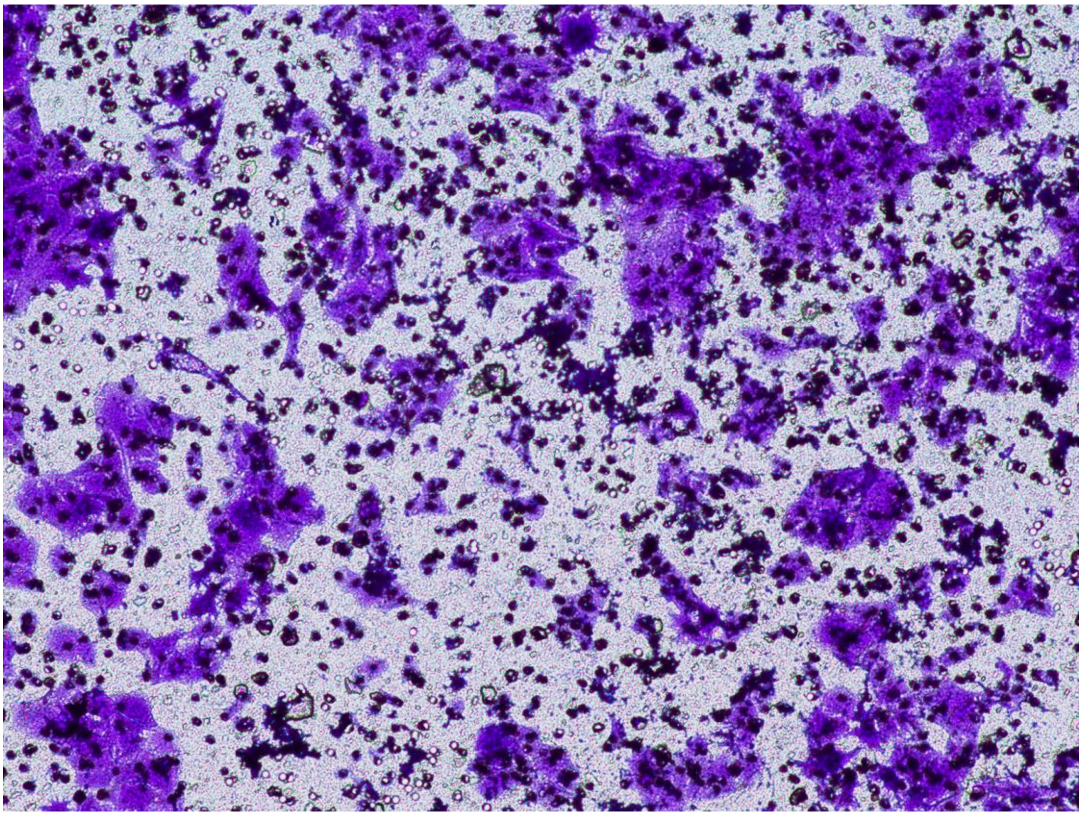

Picture 22: The second experiment of Huh7 sh-Lnc-Myd88 group

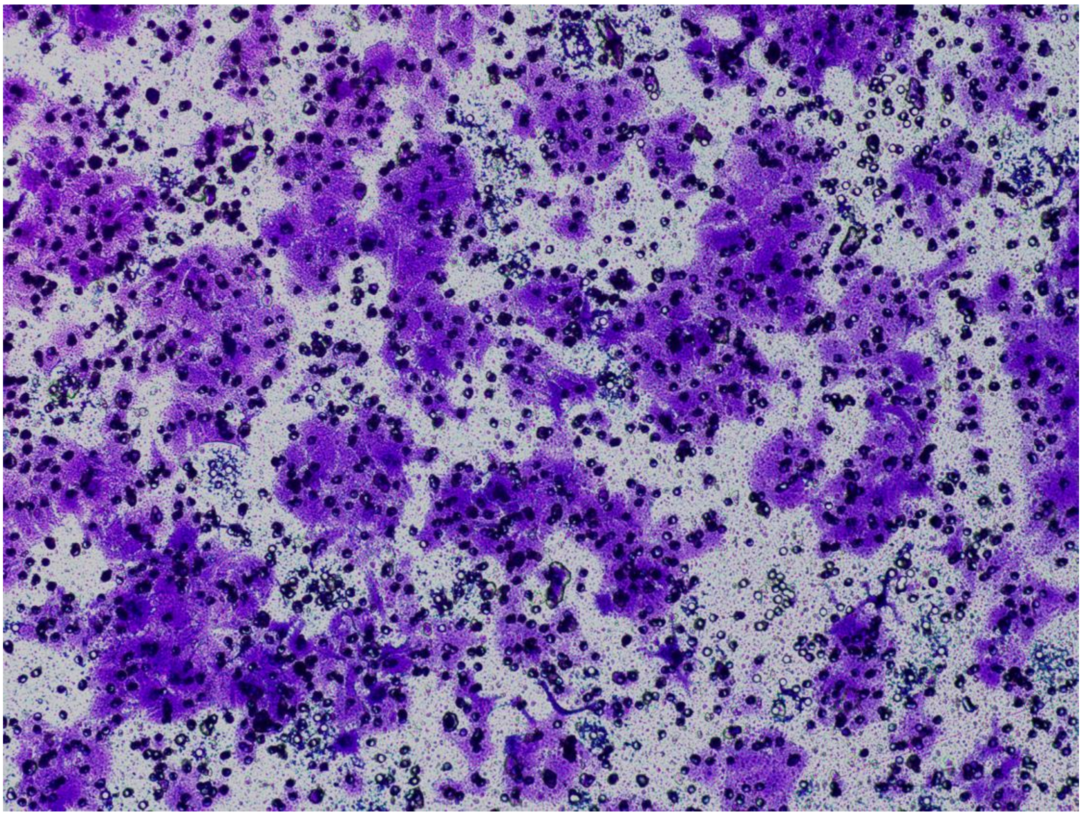

Picture 23: The third experiment of Huh7 sh-NC group

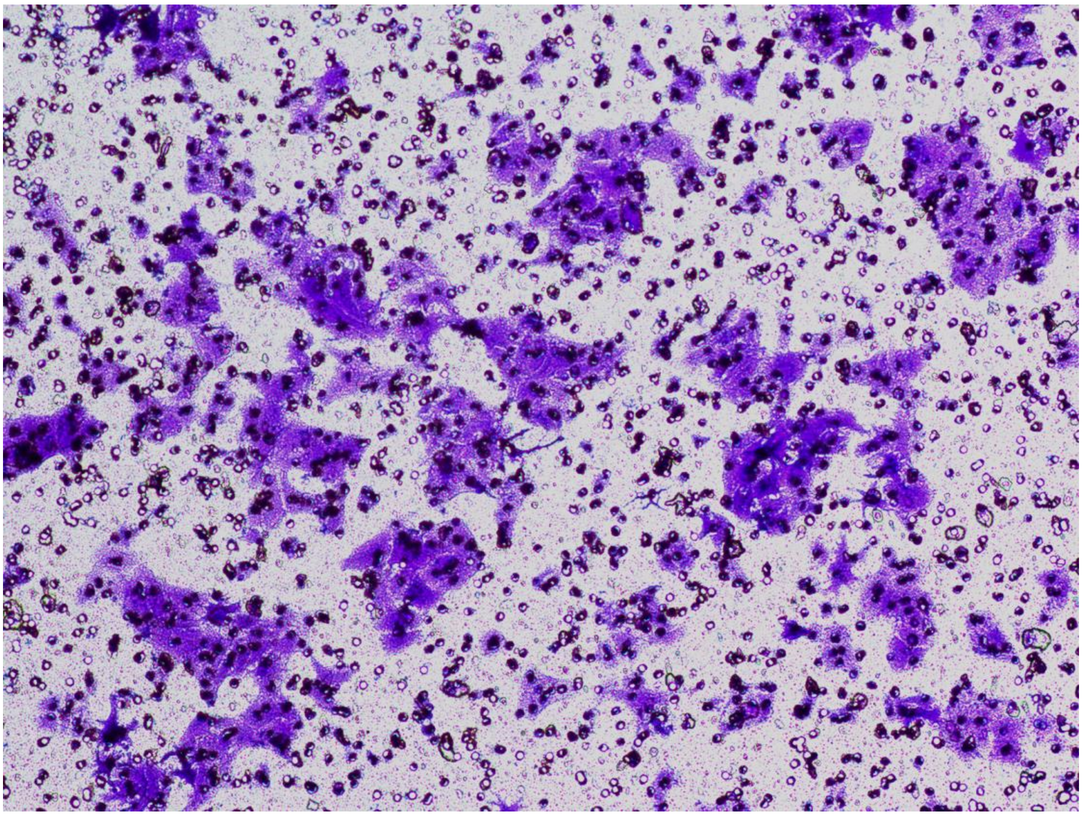

Picture 24: The third experiment of Huh7 sh-Lnc-Myd88 group
